# Genome sequencing of the neotype strain CBS 554.65 reveals the MAT1–2 locus of *Aspergillus niger*

**DOI:** 10.1186/s12864-021-07990-8

**Published:** 2021-09-21

**Authors:** Valeria Ellena, Sjoerd J. Seekles, Gabriel A. Vignolle, Arthur F. J. Ram, Matthias G. Steiger

**Affiliations:** 1 Austrian Centre of Industrial Biotechnology (ACIB GmbH), Muthgasse, 18 Vienna, Austria; 2 Institute of Chemical, Environmental and Bioscience Engineering, TU Wien, Gumpendorfer Straße 1a BH, 1060 Vienna, Austria; 3 TiFN, P.O. Box 557, 6700 AN Wageningen, The Netherlands; 4 Leiden University, Institute of Biology Leiden, Molecular Microbiology and Biotechnology, Sylviusweg 72, 2333 BE Leiden, The Netherlands

**Keywords:** Sexual development, Mating-type locus, Mitochondrial DNA, Centromere, ATCC 16888, NRRL 326

## Abstract

**Background:**

*Aspergillus niger* is a ubiquitous filamentous fungus widely employed as a cell factory thanks to its abilities to produce a wide range of organic acids and enzymes. Its genome was one of the first *Aspergillus* genomes to be sequenced in 2007, due to its economic importance and its role as model organism to study fungal fermentation. Nowadays, the genome sequences of more than 20 *A. niger* strains are available. These, however, do not include the neotype strain CBS 554.65.

**Results:**

The genome of CBS 554.65 was sequenced with PacBio. A high-quality nuclear genome sequence consisting of 17 contigs with a N50 value of 4.07 Mbp was obtained. The assembly covered all the 8 centromeric regions of the chromosomes. In addition, a complete circular mitochondrial DNA assembly was obtained. Bioinformatic analyses revealed the presence of a MAT1-2-1 gene in this genome, contrary to the most commonly used *A. niger* strains, such as ATCC 1015 and CBS 513.88, which contain a MAT1-1-1 gene. A nucleotide alignment showed a different orientation of the MAT1–1 locus of ATCC 1015 compared to the MAT1–2 locus of CBS 554.65, relative to conserved genes flanking the MAT locus. Within 24 newly sequenced isolates of *A. niger* half of them had a MAT1–1 locus and the other half a MAT1–2 locus. The genomic organization of the MAT1–2 locus in CBS 554.65 is similar to other *Aspergillus* species. In contrast, the region comprising the MAT1–1 locus is flipped in all sequenced strains of *A. niger*.

**Conclusions:**

This study, besides providing a high-quality genome sequence of an important *A. niger* strain, suggests the occurrence of genetic flipping or switching events at the MAT1–1 locus of *A. niger.* These results provide new insights in the mating system of *A. niger* and could contribute to the investigation and potential discovery of sexuality in this species long thought to be asexual.

**Supplementary Information:**

The online version contains supplementary material available at 10.1186/s12864-021-07990-8.

## Background

*Aspergillus niger* is a filamentous fungus classified in the section *Nigri* of the genus *Aspergillus.* Its versatile metabolism allows it to grow in a wide variety of environments [1]. Since the early twentieth century it has become a major industrial producer of organic acids, such as citric and gluconic acid, and enzymes, including amylases and phytases [2, 3]. The United States Food and Drug Administration has given it GRAS (Generally Regarded As Safe) status because of its long history of industrial use [3].

First genome sequencing projects were focused on industrial relevant strains. In 2007, the genome sequence of the enzyme-producing strain CBS 513.88 was published [4], followed by the sequencing of the citric acid-producing strain ATCC 1015 in 2011 [5]. At the moment, the genome sequences of 23 *A. niger* strains are available in GenBank. Surprisingly, the *A. niger* strain CBS 554.65 has not yet been sequenced although it is the official neotype strain of this species [6]. This strain was isolated from a tannic-gallic acid fermentation in Connecticut (USA) and it is listed as the (neo-)type strain by international strain collections, such as the Westerdijk Institute (CBS 554.65), the American Type Culture Collection (ATCC 16888) and the ARS Culture Collection (NRRL 326). According to the International Code of Nomenclature for algae, fungi and plants (Shenzhen Code) a neotype is “a specimen or illustration selected to serve as nomenclatural type if no original material exists, or as long as it is missing” [7]. The importance of strain CBS 554.65 lies in its use as biological model and reference strain for morphological observations and taxonomical studies. *A. niger* was previously shown to be able to form sclerotia [8–11], which are an important prerequisite for the sexual development in closely related species. In 2016 the presence of a MAT1–2 locus in the genome of CBS 554.65 was mentioned in a study [12], making this strain an interesting candidate for investigating sexuality in *A. niger*.

The MAT loci are regions of the genome which contain one or more open reading frames of which at least one encodes a transcription factor [13, 14]. Conventionally, the MAT locus containing a transcription factor with an α1 domain similar to the MATα1 of *S. cerevisiae* is called MAT1–1, while the MAT locus containing a transcription factor with a high mobility group (HMG) domain is called MAT1–2 [13]. The corresponding genes are usually called MAT1-1-1 and MAT1-2-1 [13]. The first number indicates that the two sequences are found in the same locus. Due to their sequence dissimilarities they are not termed alleles but idiomorphs [15]. MAT1-1-1 and MAT1-2-1 are major players in the sexual cycle of fungi. They contain DNA binding motifs and were shown to control the expression of pheromone and pheromone-receptor genes during the mating process [16–18]. In heterothallic species, which are self-incompatible, only one of the two MAT genes is found and mating can occur only between strains of opposite mating-type [13]. In homothallic species, which are self-fertile, both MAT genes are present, either linked or unlinked, in the same genome [19]. In the ascomycetes, the sequences flanking the MAT loci are highly conserved [13, 20, 21]. In the aspergilli, as well as in other fungi, including yeasts, the MAT idiomorphs are usually flanked by the genes *slaB*, encoding for a cytoskeleton assembly control factor, and the DNA lyase *apnB*. An anaphase promoting complex gene (*apcE*) is also sometimes present [21].

Although present in previously sequenced genomes, the second mating-type locus of *A. niger* has not been described in detail. In this study, we present the full genome sequence of a MAT1–2 *A. niger* strain and compare its MAT locus to the one of strain ATCC 1015 and those of 24 de novo sequenced *A. niger* isolates containing both MAT1–1 and MAT1–2 loci.

## Materials and methods

### Strains

The genetic organization of the MAT locus present in *A. niger* CBS 554.65 (ATCC 16888, NRRL 326) was analyzed and compared to the MAT locus of *A. niger* ATCC 1015 and 24 *A. niger* isolates obtained from the Westerdijk Fungal Biodiversity Institute (Uppsalalaan 8, Utrecht, the Netherlands). The isolates analyzed are listed in Table S1 (Additional file 1).

### Media

The morphology of strain CBS 554.65 was inspected on minimal medium [22] and malt extract agar (30 g/L malt extract (AppliChem, Darmstadt, Germany) and 5 g/L peptone from casein (Merck KGaA, Darmstadt, Germany)). The strain was 4-point inoculated and incubated at 30 °C for one week.

### Genome sequencing and annotation

The genome of the *A. niger* neotype strain CBS 554.65 was sequenced with the PacBio® technology using the PacBio SEQUEL system (Sequencing Chemistry S/P2-C2/5.0) by the Vienna Biocenter Core Facilities (VBCF). The genome was assembled with the default HGAP4 pipeline in PacBio SMRTlink version 5.1.0.26412. The mitochondrial DNA was assembled using CLC Genomic Workbench 12.0 (QIAGEN). The genome annotation of CBS 554.65 was performed with Augustus [23], by training the tool on the genome annotation of the strain ATCC 1015 as reference.

PCRs were performed on the genomic DNA of CBS 554.65 to confirm sequencing and assembly results. Primer pairs chr5_left_fwd/chr5_left_rev and chr5_right_fwd_1/chr5_right_rev_1 were used to amplify 1756 bp and 1638 bp respectively in the left and in the right region of chr5_00008F. Primers B150 and B151 were used to amplify 1644 bp in the MAT1–1 locus of ATCC 1015. Primers B151 and B152 were used to amplify 2009 bp in the MAT1–2 locus of CBS 554.65. PCR products were sequenced by Microsynth AG.

The MAT locus sequences of 24 *A. niger* isolates were extracted from the complete genome sequences obtained with the Illumina technology and assembled using SPADes [24] (data not published). Homologues of the MAT genes in these isolates were determined based on local Blastn searches using genes obtained from CBS 554.65 and ATCC 1015 as query. In 18 out of the 24 *A. niger* isolates the MAT locus was distributed over multiple scaffolds. In order to verify the location of the MAT genes and their orientation in these strains, diagnostic PCRs and subsequent sequencing were performed to fill in silico gaps within the MAT locus. Primers used in this study are listed in Table S2 (Additional file 2).

### Bioinformatic analyses

The genome and the gene set of CBS 554.65 were evaluated using Quast v5.0.2 [25, 26], which includes a benchmarking with Benchmarking Universal Single-Copy Orthologs (BUSCO) v3.0.2. This was performed with the fungal dataset of 290 BUSCOs from 85 fungal species [27]. The genome was masked using RepeatMasker v4.0.9 to identify repetitive elements [28]. Transfer RNA genes were detected using tRNAscan-SE v1.3.1 [29].

The unprocessed reads were mapped to the assembly with the Burrows-Wheeler Alignment Tool (bwa) [30, 31] and the mapping was sorted with SAMtools [32]. The average coverage based on the sorted mapping was calculated in the R environment [33]. The mappings for each individual scaffold were plotted in R and coverage graphs for each scaffold obtained.

The proteomes of the strains CBS 554.65 and NRRL3 were aligned using DIAMOND blastp [34, 35] with an E-value of e^− 10^. The output, consisting of the unique proteins of CBS 554.65 compared to NRRL3, was filtered with a blastx analysis to remove unannotated proteins and analyzed with pannzer2 [36]. The same analysis was performed on the complete proteome of strain CBS 554.65. A singular enrichment analysis (SEA) was performed on the GO term set of unique proteins of CBS 554.65 referenced to the entire GO term set of CBS 554.65 with agriGO [37, 38].

The genome sequences of strains ATCC 1015, NRRL3 and CBS 513.88 were retrieved from JGI [39]. Analyses of the position of the MAT genes within the MAT locus for *A. niger* strains were performed either on BLAST, by searching in the whole-genome shotgun contig database (wgs) of *A. niger*, or on CLC Main Workbench 20.0.2 (QIAGEN). The same analysis was performed for *A. welwitschiae* strains on BLAST against the whole-genome shotgun contig database (wgs) limited by organism (*Aspergillus niger*) and with FungiDB for the other *Aspergillus* species [40]. Sequence analyses and alignments were performed with CLC Main Workbench 20.0.2 (QIAGEN).

## Results and discussion

### Morphology of strain CBS 554.65

**Fig. 1:**
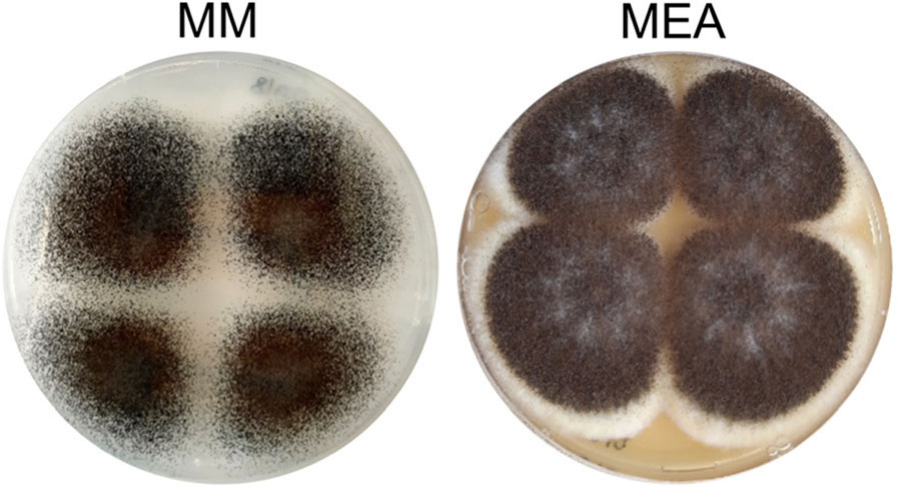
Morphology of the neotype strain CBS 554.65 on minimal medium (MM) and malt extract agar (MEA)

The strain CBS 554.65 is the *A. niger* neotype, a reference strain for morphological and taxonomical analyses. The morphology of this strain grown on minimal medium and malt extract agar can be observed in Fig. 1. On both media CBS 554.65 forms abundant conidia, black on minimal medium and dark brown on malt extract agar.

### Genome sequence and analysis

The genome sequencing of the neotype strain CBS 554.65 yielded 5.3 Gbp in 287,000 subreads. The mean length was 18.4 Kbp for the longest subreads and half of the data was in reads longer than 29 Kbp. The assembly consisted of 17 contigs with a total of 40.4 Mbp and a 127-fold coverage. Half of the size of the genome is comprised in 4 scaffolds (L50) of which the smallest has a length of 4.07 Mbp (N50). The GC content is 49.57%. 100% complete BUSCOs (Benchmarking Universal Single-Copy Orthologs) with 2 duplicated and no fragmented BUSCOs were found. The repetitive regions were identified with RepeatMasker v4.0.9 [28]. Using this approach, we were able to recognize interspersed repeats, such as long interspaced nuclear repeats (LINEs) and long terminal repeats (LTR), short interspaced nuclear repeats (SINEs), transposable element like repeats as well as small RNAs, tRNA genes, simple repeats and low complexity repeats. A total of 669,638 bp of the genome was flagged as repetitive, this represents 1.66% of the total genome. In addition, a tRNA prediction with tRNAscan-SE v1.3.1 was performed using the unmasked genome, because fungal specific SINEs were associated with tRNAs. Complete genome characteristics are reported in Tables S3 and S4 of Additional file 3.

The nuclear genome was annotated with Augustus, using the genome of strain ATCC 1015 as reference. Based on this automated annotation 12,240 protein coding genes were predicted. Table 1 shows some basic characteristics of the CBS 554.65 nuclear genome, calculated with Quast, in comparison to the characteristics of other three sequenced *A. niger* strains, CBS 513.88, ATCC 1015 and NRRL3, obtained from JGI.

**Table 1:** Comparison of the basic characteristics of the nuclear genomes of 4 different *A. niger* strains

	CBS 554.65 (This study)	CBS 513.88 [4, 5]	ATCC 1015 [5]	NRRL3 [41, 42]
Genome size (Mb)	40.42	33.98	34.85	35.25
Coverage	127x	7.5x	8.9x	10x
Number of contigs	17	471	24	15
Number of scaffolds	17	19	24	15
Scaffold N50 (Mbp)	4.07	2.53	1.94	2.81
Scaffold L50	4	6	6	5
GC content (%)	49.57	50.4	50.3	49.92
Protein-coding genes	12,240	14,097	11,910	11,846

**Fig. 2:**
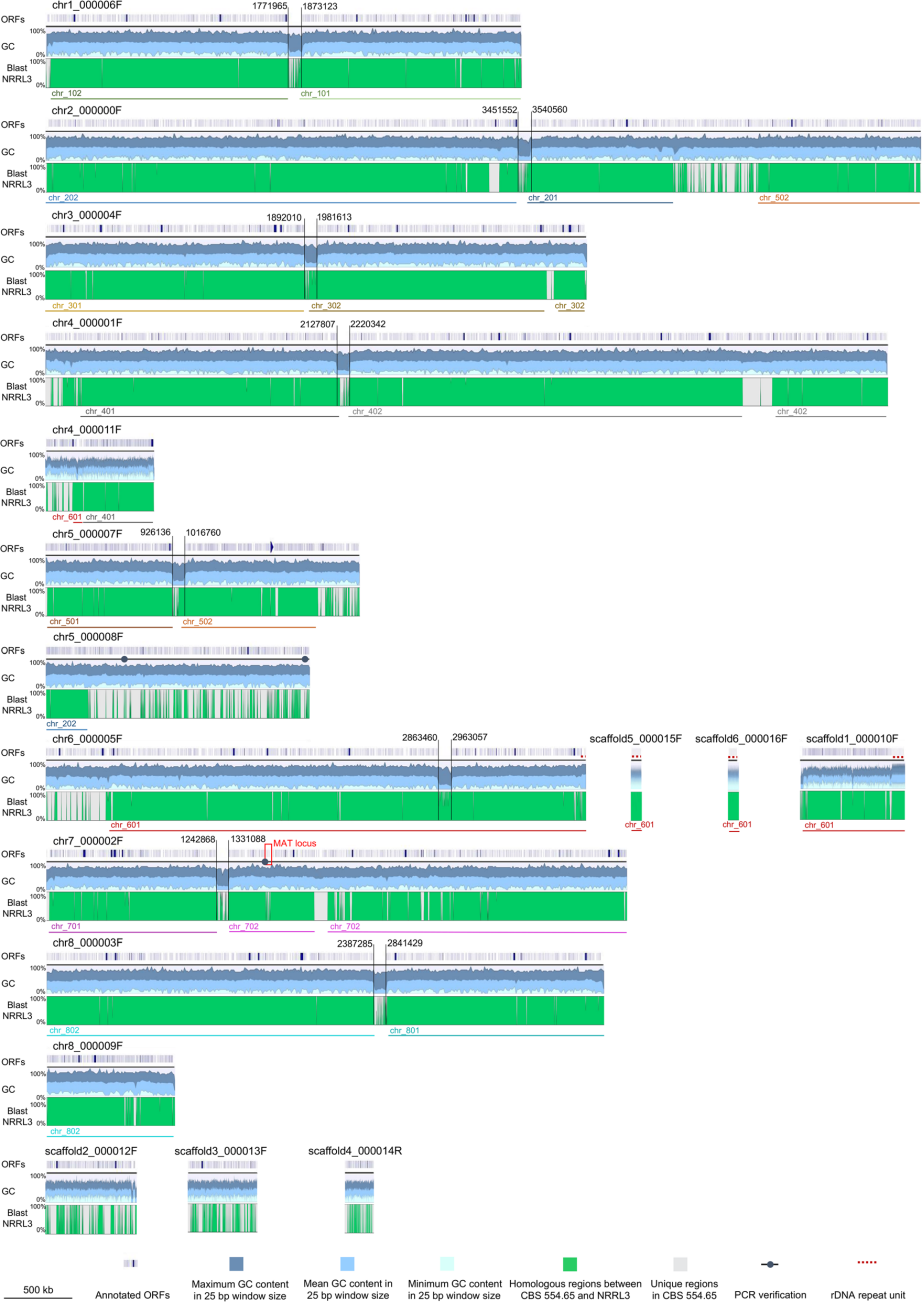
Assembly of the genome sequence of CBS 554.65 consisting of 17 contigs (in scale). For each contig (black horizontal lines) the annotated ORFs (first row), the GC content (second row) and the conservation compared to NRRL3 (third row) are schematically represented. The annotation was obtained with Augustus. The GC content was calculated using a window size of 25 bp. The upper and darker graph represents the maximum GC content value observed in that region, the middle graph represents the mean GC value and the lower graph represents the minimum GC value. The conservation graph (last row) was obtained by blasting each contig of CBS 554.65 against the whole genome of strain NRRL3. The results shown here were additionally confirmed using Mauve [47] by performing progressive alignments of each CBS 554.65 scaffold with the complete genome sequence of NRRL3 (data not shown). Green areas indicate genomic regions conserved between the two strains, grey areas indicate regions only found in CBS 554.65 and not in NRRL3. Below the conservation graph lines representing the chromosomes of strain NRRL3 are reported, as a result of the blast analysis. Chr6_00005F, scaffold1_000010F, scaffold5_000015F and scaffold6_000016F contain the highly repetitive ribosomal DNA (rDNA) gene unit, indicated with a dashed line on top of the scaffolds. Notably, for each of the 8 identified chromosomes, a centromeric region of at least 80 kb could be identified where ORFs are not annotated (indicated with two parallel and vertical lines; the first and the last nucleotide after and before the annotated ORFs, respectively, are indicated). These regions correspond to a decrease in the GC content (as indicated in the GC graph) and are only partially present in the genome of strain NRRL3 (grey areas in the blast graph). Dots on chr5_000008F and on chr7_000002F indicate the region where the PCRs were performed. The MAT locus analyzed in the following paragraphs is indicated by a red box on chromosome 7. Fig. S1 in the additional file 4 reports the comparison of the CBS 554.65 genome to the one of strain CBS 513.88. Additional information on the length of the contigs and the coordinates of the alignments are reported in Table S8 of Additional file 7

The CBS 554.65 genome assembly has an increased quality compared to the assemblies of the other strains, with a higher coverage, a higher N50 value and a lower L50 value. CBS 554.65 has a larger genome, while the GC content is similar in the 4 strains. For each of the 8 chromosomes, a putative centromeric region between 88 and 100 kb was identified, which is highlighted in Fig. 2 with vertical black lines. These regions have a GC content between 17.1 and 18.4%, significantly lower than the GC content characterizing the total genome (49.57%) and do not contain any predicted ORF. The only exception is a single ORF of 219 nucleotides in the centromere of chromosome 1. This is found in a 7 kb region of the centromere with a higher GC content compared to the GC content of the entire centromere, suggesting the presence of a mobile element. A conserved domain search [43] on this sequence gave as hits CHROMO and chromo shadow domains (accession: cd00024), ribonuclease H-like superfamily domain (accession: cl14782), integrase zinc binding domain (accession: pfam17921), reverse transcriptase domain (accession: cd01647), RNase H-like domain found in reverse transcriptase (accession: pfam17919) and a retropepsin-like domain (accession: cd00303). The presence of the last four domains suggests that the analyzed sequence has a retroviral or a retrotransposon origin. Similar sequences with domains for reverse transcriptase were also found in the centromeres of chromosomes 5, 6 and 7. Transposons and retrotransposons have been identified in the centromeres of other eukaryotes, including fungi [44, 45]. Blast analyses of the single chromosomes of strain CBS 554.65 against the complete genome of strain NRRL3 and of strains CBS 513.88 showed that the putative centromeres are almost completely lacking from the genome assembly of NRRL3 (Fig. 2, grey areas in the blast graph) and CBS 513.88 (Fig. S1, Additional file 4). Although difficult to identify, centromeric regions in filamentous fungi are composed of complex and heterogeneous AT rich sequences which can stretch up to 450 kb [45, 46]. Due to the likely presence of near-identical long repeats, centromeres are difficult to sequence and assemble [46] which explains why they are lacking in strain NRRL3. The blast analyses against NRRL3 and CBS 513.88 showed that other large regions of the genome of CBS 554.65 do not find homology in NRRL3 or in CBS 513.88. To confirm that these unique regions are not artifacts, the sequencing reads of CBS 554.65 were remapped to the genome. 298,301 reads (90.38% of the total reads) were remapped to the nuclear genome yielding an average coverage calculated on scaffold level of 127x. Figure S2 in the additional file 5 shows the coverage plots for each of the 17 contigs constituting the nuclear genome sequence. Continous coverage was also obtained for the CBS 554.65 regions not found in NRRL3 such as those present in chromosome 2 (chr2_00000F), chromosome 4 (chr4_000001F) and chromosome 5 (chr5_000008F) (Fig. S2, Additional file 5). Moreover, two analytic PCR reactions were successfully performed on the non-homologous region on chromosome 5 (chr5_000008F, Fig. 2). Sequencing of the PCR products confirmed the sequence obtained by genome assembly. The long reads and the high coverage characterizing this genome project allow to assemble sequences which are missing from previous genome assemblies obtained with other sequencing technologies. The number of protein-coding genes in CBS 554.65 is in line with what was found in ATCC 1015 and NRRL3. The large difference in the protein-coding genes in strain CBS 513.88 is likely caused by overpredictions, as previously suggested [5]. A comparison of the proteome of CBS 554.65 and NRRL3 by a blastp analysis showed that there are 694 unique protein sequences in the proteome of CBS 554.65 compared to NRRL3 (additional file 6, Table S6) and 209 unique protein sequences in the proteome of NRRL3 compared to CBS 554.65 (additional file 6, Table S7). GO terms were assigned to proteins and a GO term enrichment analysis was performed with agriGO [37, 38]. 39 GO terms were significantly enriched in the set of unique CBS 554.65 GO terms when referenced to the entire CBS 554.65 GO term set (additional file 6, Table S5, Figs. S3 and S4). Interestingly, GO terms related to thiamine, cholesterol metabolic processes as well as RNA processing are enriched. Overall, this demonstrates that in this genome sequence novel protein sequences were detected, which are absent from previous reference genome projects and might yield novel insights into the biology of this fungus.

### Mitochondrial DNA

**Fig. 3:**
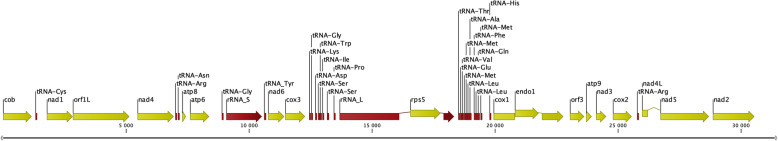
Annotation of the 31 kbp circular mtDNA sequence (displayed in a linear projection): ORF (yellow), rRNA, tRNA (red)

The mitochondrial DNA is often neglected in genome projects, which tend to focus on the nuclear genome. In *A. niger* only one mitochondrial DNA (mtDNA) assembly was reported, for the strain N909 [48]. In this study, the mtDNA of strain CBS 554.65 was de novo assembled from PacBio reads as a circular DNA with a length of 31,363 bp. MtDNA is abundant in whole genome sequencing projects and the read coverage of the assembly (average: 1220 x, min: 328 x, max: 1674 x) is thus higher than that for the nuclear genome. In total 18 ORFs, 26 tRNA and 2 rRNA sequences were annotated (Fig. 3). All 15 core mitochondrial genes reported for *Aspergillus* species were identified with a similar gene organization [49]. In addition, three accessory genes *orf1L*, *orf3* and *endo1* were annotated. The gene *endo1* is located in the intron of *cox1* and encodes a putative homing endonuclease gene belonging to the LAGLIDADG family frequently found in the *cox1* intron of other filamentous fungi [49]. The gene *orf3* encodes a hypothetical protein of 191 residues, which is also present in the mtDNA of strain N909 but was not annotated there. Surprisingly this unknown protein has a good hit against an unknown protein of *Staphylococcus aureus* (99% identity, WP_117225298.1), however not against other proteins of *Aspergillus* species. In *A. niger* strain N909 two other unknown proteins are encoded in *orf1* and *orf2*. These two open reading frames are connected to *orf1L* in *A. niger* CBS 554.65 yielding a potential protein product with 739 amino acid residues. This is similar to an open reading frame located at the same position between *nad1* and *nad4* in the mtDNA of *A. flavus* NRRL 3357 (AFLA_m0040), with a size of 667 amino acid residues. In the N-terminal region of both putative proteins, transmembrane spanning regions can be predicted supposing a location in a mitochondrial membrane. However the C-terminal regions are not conserved between *A. niger* and *A. flavus* proteins. We suggest to use the mitochondrial assembly of CBS 554.65 as a reference sequence for *A. niger* mitochondria because it is known that strain N909 is resistant to oligomycin [50]. This resistance is typically linked to mutations in the mtDNA, either in *atp6* [51] or *atp9* [52], and indeed two mutations are found in *atp6* of strain N909 (L26W and S173L).

### Discovery and sequencing of a MAT1–2 *A. niger* strain

The genome sequencing and analysis of strain CBS 554.65 allowed to determine the mating-type of this strain. The sequence of the putative MAT1-2-1 gene (g9041) was searched in the standard nucleotide collection database (nr/nt) using Blastn. This gave as hits the mating-type HMG-box protein MAT1-2-1 of other aspergilli, including *A. neoniger* (with an identity of 93.25%) and *A. tubingensis* (with an identity of 93.07%). As such, we consider gene g9041 to be homologous to the MAT1-2-1 gene of other *Aspergillus* species.

This is in line with a previous study that showed the presence of a MAT1-2-1 sequence in the CBS 554.65 strain through a PCR approach [12]. Here we report the complete genome sequence of an *A. niger* strain having a MAT1-2-1 gene. The availability of this genome sequence represents an important tool for further studies investigating the sexual potential of *A. niger*. The presence of both opposite mating-type genes in different strains belonging to the same species represents a strong hint of a sexual lifestyle [14].

### MAT1–2 locus analysis and comparison to MAT1–1

**Fig. 4:**
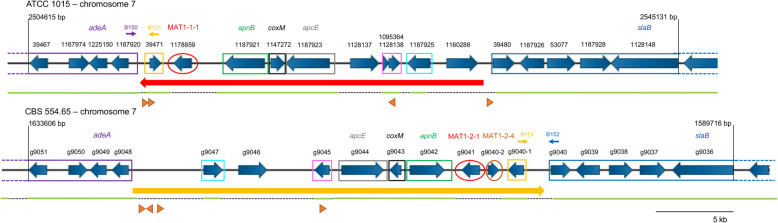
Nucleotide alignment between the same genomic region of ATCC 1015 (MAT1–1) and CBS 554.65 (MAT1–2). Genes found in both strains are indicated with a box of the same color, MAT genes are indicated with a circle and unmarked genes are unique in each strain*.* Below each genomic region, green lines indicate regions homologous in the two strains and dotted lines regions unique for each strain. A red arrow indicates the genomic region of ATCC 1015 which contains the MAT1-1-1 gene and appears flipped compared to the corresponding region in CBS 554.65 (yellow arrow). Small arrows with numbers B150, B151 and B152 indicate primers used for PCRs. Orange triangles indicate the presence of a 7 bp motif (5′-TTACACT)

The locus of strains CBS 554.65 containing the MAT1-2-1 gene was compared in silico to the locus of strain ATCC 1015 containing the MAT1-1-1 gene. This was done to determine whether the genes flanking the MAT1-1-1 gene are also present in the genome of the MAT1–2 strain and vice versa. A region of 40,517 bp, spanning from gene Aspni7|39467 (genomic position 2,504,615 in the v7 of the ATCC 1015 genome) to gene Aspni7|1128148 (genomic position 2,545,131) was aligned to the corresponding region of strain CBS 554.65 (Fig. 4). In CBS 554.65 the two genes homologous to Aspni7|39467 (g9051) and Aspni7|1128148 (g9036) are comprised in a sequence of 43,891 bp, almost 4 kb longer than in ATCC 1015. The identifiers of the genes included in these regions are indicated in Fig. 4 and additionally reported in Table 2, with their predicted function retrieved from FungiDB or blast analysis. The alignment shows that the MAT genes occupy the same genomic location at chromosome 7. The genes comprised in the analyzed loci are mostly conserved between the two strains, with the exception of genes Aspni7|1178859 (MAT1-1-1), Aspni7|1128137 and Aspni7|1160288, unique for ATCC 1015, and g9046, g9041 (MAT1-2-1) and g9040–2 (MAT1-2-4), unique for CBS 554.65. Aspni7|1128137 has predicted metal ion transport activity and it is found in other *Aspergillus* species, either heterothallic with a MAT1-1-1 or a MAT1-2-1 gene or homothallic. It is not found near the MAT gene, with the exception of *A. brasiliensis* and *A. ochraceoroseus*. Aspni7|1160288 has a domain with predicted role in proteolysis and its homolog in other aspergilli is present at another genomic locus, not in proximity to the MAT gene. A homolog of gene g9046 was found by Blastn search in *Aspergillus vadensis*, in a different location of the genome than the MAT locus. These results suggest that these unique genes are unlikely to be part of the “core” MAT locus. The gene g9040–2 is a putative homolog of the MAT1-2-4 gene in *A. fumigatus*, an additional mating-type gene required for mating and cleistothecia formation [53]. Another difference between ATCC 1015 and CBS 554.65 is represented by the gene putatively encoding for a HAD-like protein. While this gene is complete in CBS 554.65 (g9045), it appears disrupted in ATCC 1015 and, therefore, doubly annotated in this strain (Aspni7|1095364 and Aspni7|1128138). The other genes present in the selected genomic region show a high level of conservation, with a higher synteny further away from the MAT genes (genes in the purple and blue boxes). Moreover, genes encoding for the DNA lyase a*pnB*, the cytoskeleton control assembly factor *slaB* and the anaphase promoting complex *apcE* are present in both MAT loci. These genes are normally found in the MAT loci of other fungi, including yeast [21]. Their presence in the MAT loci of *A. niger* further confirms the high level of conservation characterizing this locus. In heterothallic ascomycetes the MAT genes are commonly included between the genes *apnB* and *slaB* [21]. From the alignment in Fig. 4 the relative position of the MAT genes to *apnB* and *slaB* can be analyzed. In CBS 554.65 the MAT1-2-1 gene (g9041) is flanked by *apnB* and *slaB* respectively upstream and seven genes downstream. In contrast, in the MAT1–1 locus of strain ATCC 1015 the MAT gene is flanked downstream by *apnB* and upstream by a conserved sequence including *adeA*, while *slaB* is found on the same side of *apnB*. The entire genomic locus, containing the MAT1-1-1 gene and eight other genes (23 kbp indicated by the red arrow in Fig. 4), shows a flipped orientation compared to the corresponding locus in CBS 554.65 containing the MAT1-2-1 gene (indicated by an orange arrow in Fig. 4). The ORF direction of the conserved genes *apnB*, *coxM* and *apcE* additionally confirms the different orientation of this locus in the two strains. In addition, PCRs performed with primers B150, B151 and B152 (Fig. 4) yielded expected bands, confirming the orientation of the MAT loci of both ATCC 1015 and CBS 554.65. By sequence analysis, a repetitive 7 bp DNA motif (5´-TTACACT) was found in the MAT1–1 locus (orange triangles in Fig. 4), where the homology between the MAT1–1 and MAT1–2 loci breaks (in proximity to *adeA* and *slaB*). An additional site of this motif was found in the gene encoding a HAD-like hydrolase (Aspni7|1128138). This motif is present at similar positions in at least two other sequenced MAT1–1 strains of *A. niger* (N402, CBS 513.88). Differently, the MAT1–2 strain presents this motif only at the site close to the *adeA* gene and in the putative HAD-like hydrolase gene (g9045), but not at the site close to the *slaB* gene.

**Table 2:** List of genes included in the genomic region comprising the MAT genes

ATCC 1015	CBS 554.65	Predicted function retrieved from FungiDB or blast
Aspni7|39467	g9051	Hypothetical protein
Aspni7|1167974	g9050	CIA30-domain containing protein – Ortholog(s) have role in mitochondrial respiratory chain complex I assembly
Aspni7|1225150	g9049	SAICAR synthetase (*adeA*)
Aspni7|1187920	g9048	Homolog in CBS 513.88 has domain(s) with predicted catalytic activity, metal ion binding, phosphoric diester hydrolase activity
Aspni7|39471	g9040–1	Hypothetical protein
Aspni7|1178859	–	Mating-type protein MAT1-1-1
Aspni7|1187921	g9042	DNA lyase Apn2|Hypothetical protein
Aspni7|1147272	g9043	Hypothetical cytochrome C oxidase|Mitochondrial cytochrome c oxidase subunit VIa
Aspni7|1187923	g9044	Ortholog(s) are anaphase-promoting complex proteins
Aspni7|1128137	–	Homolog in CBS 513.88 has domain(s) with predicted metal ion transmembrane transporter activity, role in metal ion transport, transmembrane transport and membrane localization
Aspni7|1095364	g9045	HAD-like protein; Homolog in CBS 513.88 has domain(s) with predicted hydrolase activity
Aspni7|1128138	g9045	HAD-like protein; Homolog in CBS 513.88 has domain(s) with predicted hydrolase activity
Aspni7|1187925	g9047	Glycosyltransferase Family 8 protein - Ortholog(s) have acetylglucosaminyltransferase activity, role in protein N-linked glycosylation and Golgi medial cisterna localization
Aspni7|1160288	–	Aspartic protease|Hypothetical aspartic protease
Aspni7|39480	g9040	WD40 repeat-like protein
Aspni7|1187926	g9039	Aldehyde dehydrogenase
Aspni7|53077	g9038	CoA-transferase family III
Aspni7|1187928	g9037	Salicylate hydroxylase
Aspni7|1128148	g9036	Cytoskeleton assembly control protein Sla2
–	g9046	Hypothetical protein
–	g9041	Mating-type HMG-box protein MAT1-2-1
–	g9040–2	Hypothetical protein – Putative homologue of MAT1–2-4 of *A. fumigatus*

Methods to identify the opposite mating-type in strains isolated from natural sources often rely on the use of primers designed to bind to *apnB* and *slaB*, since these are the genes that commonly flank the MAT gene itself [54, 55]. In both mating-type *A. niger* strains, *slaB* is found more than 12 kbp away from the MAT gene. In addition, the relative orientation of *apnB* to *slaB* is different in strains having opposite mating types. This might explain why the MAT1–2 locus was only mentioned by one previous study [12] but never described in detail so far.

Both the particular orientation of the MAT locus and the presence of a repetitive motif in the MAT loci suggest that a genetic switch or a flipping event might have occurred or is still ongoing in *A. niger*, which might affect the expression of the MAT genes. Genetic switching events at the MAT locus are known for other ascomycetes, particularly yeasts. For instance, in *S. cerevisiae* a switching mechanism involving an endonuclease and two inactive but intact copies of the MAT genes allows to switch the MAT type of the cell [56]. Expression of the MAT gene is instead regulated in the methylotrophic yeasts *Komagataella phaffii* and *Ogataea polymorpha* via a flip/flop mechanism [57, 58]. In these species, a 19 kbp sequence including both mating type genes is flipped so that a MAT gene will be close to the centromere (5 kbp from the centromere) and, therefore, silenced while the other will be transcribed. In CBS 554.65 the region comprising the MAT1-2-1 gene is present at around 280 kbp downstream of the putative centromere, which is much further away of what observed for *K. phaffi* and *O. polymorpha*. However, in certain basidiomycetes, such as *Microbotryum saponariae* and *Microbotryum lagerheimii*, the mating-type locus HD (containing the homeodomain genes) is around 150 kbp distant from the centromere and linked to it [59]. It was proposed that the proximity to the centromere in these species might be enough to reduce recombination events [59]. The effect of the distance between the centromere and the MAT genes in *A. niger* merits further attention, especially in view of a potential sexual cycle characterizing this species.

Inversion at the MAT locus have been described for certain homothallic filamentous fungi such as *Sclerotinia sclerotiorum* and *Sclerotinia minor* [60, 61]. Field analysis of a large number of isolates showed that strains belonging to these species can either present a non-inverted or an inverted MAT locus. In the inverted orientation two of the four MAT genes at the locus have the opposite orientation and one gene is truncated. In the case of *S. sclerotiorum*, differences in the gene expression were observed between inverted and non-inverted strains. This inversion, induced by crossing-over between two identical inverted repeat present in the locus, likely happens during the sexual cycle before meiosis [60]. The analysis of a larger number of *A. niger* isolates is required to investigate whether opposite orientations of both MAT loci exist for this species as well and what the implications of such inversions might be. Chromosomal inversions are considered to prevent recombination between sex determining genes in higher eukaryotes, such as animals and plants [62]. Further studies are required to investigate whether *A. niger* possesses a genetic switching mechanism controlling its sexual development.

### Genetic comparison of MAT loci in different aspergilli and additional *A. niger* strains

This study revealed a particular configuration for the MAT1–1 locus of strain ATCC 1015. For this reason, the orientation of the MAT locus of additional *Aspergillus* species for which a genome sequence is available was analyzed. Firstly, the genes *adeA* and *slaB* were retrieved as they are conserved and often found at the right and left flank of the MAT gene, respectively (Fig. 4). Subsequently, the position of the MAT gene was compared to the three conserved genes *apnB*, *coxM* and *apcE*. The MAT gene could be either included between *adeA* and *apnB*, like in ATCC 1015 (flipped position), or between *apnB and slaB*, like in CBS 554.65 (conserved position). The results of this analysis are reported in Table 3. A complete table with the identifiers of all genes analyzed is reported in the Additional file 8.

**Table 3:** MAT gene identifiers of the analyzed *Aspergillus* strains and their position in the MAT locus

Section	Species	Strain	Mating-type gene - MAT	Mating-type	MAT position	Sexual cycle described for the species
*Nigri*	*A. welwitschiae*	CBS 139.54	172,181	MAT1–1	flipped	No
	*A. kawachii (A. luchuensis)*	IFO 4308	AKAW_03832	MAT1–2	conserved	No
	*A. luchuensis*	106.47	ASPFODRAFT_180958	MAT1–1	conserved	No
	*A. tubingensis*	G131	Not annotated	MAT1–2	conserved	Yes [63]
		CBS 134.48	ASPTUDRAFT_124452	MAT1–1	conserved	
	*A. niger*	CBS 554.65	g9041	MAT1–2	conserved	No
		ATCC 1015	ASPNIDRAFT2_1178859	MAT1–1	flipped	
	*A. brasiliensis*	CBS 101740	ASPBRDRAFT_167991	MAT1–2	flipped	No
	*A. carbonarius*	ITEM 5010	ASPCADRAFT_1991	MAT1–2	conserved	No
	*A. aculeatus*	ATCC 16872	ASPACDRAFT_1867751	MAT1–2	conserved	No
*Nidulantes*	*A. versicolor*	CBS 583.65	ASPVEDRAFT_82222	MAT1–2	conserved	No
	*A. sydowii*	CBS 593.65	ASPSYDRAFT_87884	MAT1–2	conserved	No
*Ochraceorosei*	*A. ochraceoroseus*	IBT 24754	P175DRAFT_0477739	MAT1–1	conserved	No
*Flavi*	*A. flavus*	NRRL 3357	AFLA_103210	MAT1–1	conserved	Yes [64]
	*A. oryzae*	BCC7051	OAory_01101300	MAT1–2	conserved	No
		RIB40	AO090020000089	MAT1–1	conserved	
*Circumdati*	*A. steynii*	IBT 23096	P170DRAFT_349471	MAT1–2	conserved	No
*Candidi*	*A. campestris*	IBT 28561	P168DRAFT_313902	MAT1–1	conserved	No
			P168DRAFT_285957	MAT1–2	conserved	
*Terrei*	*A. terreus*	NIH2624	ATEG_08812	MAT1–1	conserved	Yes [65]
*Fumigati*	*A. novofumigatus*	IBT 16806	P174DRAFT_462167	MAT1–2	conserved	No
	*A. fischeri*	NRRL 181	NFIA_071100	MAT1–1	conserved	Yes [66]
			NFIA_024390	MAT1–2	conserved	
	*A. fumigatus*	Af293	Afu3g06170	MAT1–2	conserved	Yes [67]
		A1163	AFUB_042900	MAT1–1	conserved	
			AFUB_042890	MAT1–2	conserved	
*Clavati*	*A. clavatus*	NRRL1	ACLA_034110	MAT1–1	conserved	Yes [68]
			ACLA_034120	MAT1–2	conserved	
*Aspergillus*	*A. glaucus*	CBS 516.65	ASPGLDRAFT_89185	MAT1–1	n.a.^1^	Yes [69, 70]
*Cremei*	*A. wentii*	DTO 134E9	ASPWEDRAFT_184745	MAT1–2	conserved	No

^1^ Conserved genes not in the MAT locus

Table 4 MAT genes which are found between *apnB* and *slaB* are considered to have a “conserved” position, while MAT genes identified between *adeA* and *apnB* are considered as “flipped”. *Aspergillus* species are grouped in sections based on the most updated classification [71]. For each species it is indicated if a sexual cycle has been reported in the literature.

**Table 4:** Mating-type and MAT gene position of the analyzed *A. niger* strains. 48 *A. niger* strains have been analyzed in respect to their MAT locus configuration. Newly sequenced *A. niger* isolates and CBS 554.65 are reported in rows above the dashed line. Among these, 12 have a MAT1–1 and 13 a MAT1–2 locus. Previously sequenced *A. niger* strains are reported in rows below the dashed line. Among these, a bias towards MAT1–1 strains is present. All the MAT1–1 strain have a flipped orientation of the MAT locus and all the MAT1–2 strains a conserved one. *MAT locus distributed over multiple scaffolds which could not be combined

MAT1–1				MAT1–2			
*A. niger* strain	Isolation source	MAT position	GenBank accession	*A. niger* strain	Isolation source	MAT position	GenBank accession
CBS 112.32	Unknown, Japan	flipped	MW809488	CBS 554.65	Tannin-gallic acid fermentation, USA	conserved	PRJNA715116
CBS 147371	Green coffee bean, India	flipped	MW809493	CBS 113.50	Leather, unknown	conserved	MW809487
CBS 147320	Grape, Australia	flipped	MW809494	CBS 124.48	Unknown	conserved	MW809489
CBS 147345	Unknown, USA	flipped	MW809501	CBS 118.52	Unknown	conserved	Incomplete coverage*
CBS 147347	Petridish, soft drink factory, The Netherlands	flipped	MW809503	CBS 147321	Arctic soil, Norway	conserved	MW809495
CBS 769.97	Leather, Unknown	flipped	MW809504	CBS 147322	Coffee, Brazil	conserved	MW809496
CBS 115989	Unknown	flipped	MW809505	CBS 147323	Raisin, Turkey	conserved	MW809497
CBS 147352	Air next to bottle blower, Mexico	flipped	MW809506	CBS 147324	Unknown	conserved	MW809498
CBS 147353	Food factory of Sanquinetto, Italy	flipped	MW809507	CBS 147482	Surface water, Portugal	conserved	Incomplete coverage*
CBS 115988	Unknown	flipped	MW809491	CBS 147344	Coffee beans, Thailand	conserved	MW809499
CBS 131.52	Leather, unknown	flipped	MW809490	CBS 133816	Black pepper, Denmark	conserved	MW809500
CBS 147343	Coffee bean, Thailand	flipped	MW809508	CBS 147346	CF patient material, The Netherlands	conserved	MW809502
H915–1	Soil, China	flipped	PRJNA288269	CBS 630.78	Army equipment, South Pacific Islands	conserved	MW809492
L2	Soil, China	flipped	PRJNA288269	RAF106	Pu′er tea, China	conserved	PRJNA503751
LDM3	Industrial production, China	flipped	PRJNA562509	3.316	Laboratory, China	conserved	PRJNA597564
FDAARGOS_311	USA	flipped	PRJNA231221	An76	Soil, China	conserved	PRJDB4313
N402 (ATCC 64974)	Laboratory, The Netherlands	flipped	PRJEB21769	JSC-093350089	ISS environmental surface, USA	conserved	PRJNA355122
ATCC 10864	Soil, Peru	flipped	PRJNA300350	MOD1-FUNGI2	Red seedless grapes, USA	Genes in different scaffolds	PRJNA482816
F3_1F3_F	ISS environmental surface, USA	flipped	PRJNA667181				
F3_4F2_F	ISS environmental surface, USA	flipped	PRJNA667181				
F3_4F1_F	ISS environmental surface, USA	flipped	PRJNA667181				
DSM 1957	Soil, France	flipped	PRJNA566102				
FGSC A1279	Laboratory, The Netherlands	flipped	PRJNA255851				
A1	Soil, China	flipped	PRJNA288269				
ATCC 1015	USA	flipped	PRJNA15785				
ATCC 13496	Soil, USA	flipped	PRJNA209543				
CBS 101883 (*A. lacticoffeatus*)	Coffee beans, Sumatra	flipped	PRJNA479910				
CBS 513.88	Unknown	flipped	PRJNA19275				
SH-2	Soil, China	flipped	PRJNA196564				
ATCC 13157 (*A. phoenicis*)	Whole shelled corn	flipped	PRJNA209548				

In the analyzed *Aspergillus* sequences the MAT gene (either MAT1-1-1 or MAT1-2-1) was mostly found between the genes *apnB* and *slaB*, such as in CBS 554.65 (conserved). The only exceptions, showing a configuration similar to the MAT1–1 locus of ATCC 1015, were the MAT1-1-1 gene of *A. welwitschiae* and the MAT1-2-1 gene of *A. brasiliensis*. This analysis could not be performed on the MAT1–2 locus of *A. welwitschiae* nor on the MAT1–1 locus of *A. brasiliensis*, due to the unavailability of sequences for strains of the opposite mating type. Seven of the analyzed species, including the closely related *A. tubingensis*, were reported to have a sexual cycle. A conserved position of the MAT gene was observed for all of these species with the exception for *A. glaucus*, whose conserved genes were not found in the vicinity of the MAT gene. These observations suggest that the position of the MAT gene and the orientation of the locus might have an impact on the sexual development of the respective fungus.

Since the orientation observed for the MAT1–1 locus of ATCC 1015 might be peculiar for this *A. niger* strain only, additional genome sequences were analyzed to determine the orientation of the MAT locus of other sequenced strains of *A. niger* (Table 4). 18 out of 23 *A. niger* strain sequences deposited in GenBank contain a MAT1-1-1 gene and they all show the same orientation of the MAT locus as observed in ATCC 1015. The other 5 strains contain a MAT1–2 locus and they all show the same conserved orientation as observed in the strain CBS 554.65. The orientation could not be determined for one MAT1–2 strain, MOD1FUNGI2, since the different analyzed genes are present in different scaffolds in the available genome sequence. Overall, 80% of the sequenced strains contain a MAT1–1 locus. The selection procedure of strains for whole-genome sequencing might be biased by their industrial relevance and might not resemble the mating-type distribution in the environment. Therefore, 24 randomly picked isolates of *A. niger* were sequenced and the MAT loci analyzed: 12 contain the MAT1–1 locus and 12 the MAT1–2 locus (Table 4).

The MAT locus configuration of these strains is similar to the configuration of strain ATCC 1015, in the case of the MAT1–1 strains, and to CBS 554.65, in the case of at least 10 out of 12 MAT1–2 strains. In the two remaining MAT1–2 strains (CBS 118.52 and CBS 147482) a gap between two genomic scaffolds could not be closed by PCR. This is likely due to the presence of a region with multiple G repeats. However, when the two separate scaffolds of these isolates were aligned to the MAT1–2 locus of CBS 554.65, they appeared to have the same locus configuration as the other 10 MAT1–2 isolates. Similarly to what was observed for ATCC 1015 and CBS 554.65, the HAD-like protein encoding gene appears disrupted in all the MAT1–1 isolates and complete in all the MAT1–2 isolates. Further studies are required to investigate whether the disruption of this gene in the MAT1–1 strains plays a role in the context of fungal development. Overall, the MAT1–1 configuration described in Fig. 4 is a peculiar feature of *A. niger* and its close relative *A. welwitschiae*. Despite the unusual orientation, the presence of a 50:50 ratio of MAT1–1:MAT1–2 among 24 randomly selected *A. niger* isolates is remarkable and suggests that sexual reproduction is occurring in this species. Interestingly, MAT1–1 occurs at higher frequency in commonly used industrial and laboratory strains. This could be pure coincidence, but it could also indicate a phenotypic difference between strains with opposite matingtypes.

## Conclusions

The *A. niger* neotype strain CBS 554.65 has now a high quality genome sequence, which covers all the 8 centromeres and includes a complete mtDNA sequence. This sequence represents an important tool for further studies. The analysis of this genome revealed the presence of a second mating-type locus (MAT1–2) in this strain, making it a suitable reference strain to investigate fungal development in *A. niger*. The position and the orientation of the MAT1-2-1 gene of all the 15 MAT1–2 *A. niger* strains analyzed was found to be similar to that of other aspergilli, with the MAT gene included between the genes *apnB* and *slaB*. The unusual orientation of the MAT1-1-1 locus found in the already sequenced *A. niger* strains and in other 12 newly sequenced isolates indicates that flipping or switching events have occurred at the MAT locus. Further research is required to investigate whether this difference in the position of the MAT genes in the opposite mating-type strains could have an effect on the expression of the genes included in this genomic region. These flipping events might have a direct impact on the sexual development in *A. niger*.

## Supplementary Information

**Additional file 1: Table S1.***Aspergillus niger* strains used in this study.

**Additional file 2: Table S2.** List of primers used in this study.

**Additional file 3: Table S3.** Genome characteristics and found Benchmarking Universal Single-Copy Orthologues (BUSCO) genes of the assembled *Aspergillus niger* strain CBS 554.65. **Table S4.** Masked repetitive elements found with RepeatMasker v4.0.9 and tRNA genes found by tRNAscan-SE v1.3.1.

**Additional file 4: Fig. S1.** Assembly of the genome sequence of CBS 554.65 consisting of 17 contigs (in scale). For each contig (black horizontal lines) the annotated ORFs (first row), the GC content (second row) and the conservation compared to CBS 513.88 (third row) are schematically represented.

**Additional file 5: Fig. S2.** Coverage plots of the scaffolds obtained by remapping the reads to the CBS 554.65 genome assembly.

**Additional file 6: Table S5.** GO term enrichment analysis of the unique GO term set of CBS 554.65 referenced to the entire GO term set of CBS 554.65. The unique CBS 554.65 proteins compared to NRRL3 are 694, of which 176 had at least one GO term assigned. **Fig. S3.** GO term enrichment analysis of the unique GO term set of CBS 554.65 referenced to the entire GO term set of CBS 554.65, assigned to the biological process ontology. **Fig. S4.** GO term enrichment analysis of the unique GO term set of CBS 554.65 referenced to the entire GO term set of CBS 554.65, assigned to the molecular function ontology. **Table S6.** Unique protein sequences in the proteome of CBS 554.65 compared to NRRL3 by a blastp analysis. **Table S7.** Unique protein sequences in the proteome of NRRL3 compared to the entire proteome of CBS 554.65 by a blastp analysis.

**Additional file 7: Table S8.** Lenght of the contigs of CBS 554.65 and coordinates of the NRRL3 and CBS 513.88 contig alignments to CBS 554.65.

**Additional file 8: Table S9.** Gene identifiers of the analyzed *Aspergillus* strains and their position in the MAT locus.

## Data Availability

This Whole Genome Shotgun project has been deposited at DDBJ/ENA/GenBank under the bioproject PRJNA715116 (accession JAGRPH000000000) [https://www.ebi.ac.uk/ena/browser/view/PRJNA715116]. The version described in this paper is version JAGRPH010000000. The genome reads of strain CBS 554.65 are available in the European Nucleotide Archive (ENA) at EMBL-EBI under accession numbers PRJEB42544 [https://www.ebi.ac.uk/ena/browser/view/PRJEB42544]. The mitochondrial genome of strains CBS 554.65 has been deposited at GenBank under the accession MW816869 [https://www.ncbi.nlm.nih.gov/nuccore/MW816869.1]. The MAT loci sequences of the *A. niger* isolates have been deposited at GenBank under the accessions: MW809487-MW809508. [https://www.ncbi.nlm.nih.gov/nuccore/MW809487, https://www.ncbi.nlm.nih.gov/nuccore/MW809488, https://www.ncbi.nlm.nih.gov/nuccore/MW809489, https://www.ncbi.nlm.nih.gov/nuccore/MW809490, https://www.ncbi.nlm.nih.gov/nuccore/MW809491, https://www.ncbi.nlm.nih.gov/nuccore/MW809492, https://www.ncbi.nlm.nih.gov/nuccore/MW809493, https://www.ncbi.nlm.nih.gov/nuccore/MW809494, https://www.ncbi.nlm.nih.gov/nuccore/MW809495, https://www.ncbi.nlm.nih.gov/nuccore/MW809496, https://www.ncbi.nlm.nih.gov/nuccore/MW809497, https://www.ncbi.nlm.nih.gov/nuccore/MW809498, https://www.ncbi.nlm.nih.gov/nuccore/MW809499, https://www.ncbi.nlm.nih.gov/nuccore/MW809500, https://www.ncbi.nlm.nih.gov/nuccore/MW809501, https://www.ncbi.nlm.nih.gov/nuccore/MW809502, https://www.ncbi.nlm.nih.gov/nuccore/MW8094503, https://www.ncbi.nlm.nih.gov/nuccore/MW8094504, https://www.ncbi.nlm.nih.gov/nuccore/MW809505, https://www.ncbi.nlm.nih.gov/nuccore/MW809506, https://www.ncbi.nlm.nih.gov/nuccore/MW809507, https://www.ncbi.nlm.nih.gov/nuccore/MW809508].
