# Supplementary material for: Genome sequencing of the neotype strain CBS 554.65 reveals the MAT1–2 locus of Aspergillus niger
Source: BMC Genomics. 2021 Sep 21;22:679. doi: 10.1186/s12864-021-07990-8 (PMC8454179; doi:10.1186/s12864-021-07990-8)
Supplement: Supplementary file 1 — Additional file 1: Table S1.Aspergillus niger strains used in this study. [file 12864_2021_7990_MOESM1_ESM.pdf]

*Table S1. Aspergillus niger strains used in this study. All the strains were obtained from the Westerdijk Fungal Biodiversity Institute. The complete genome of CBS 554.65 and the nucleotide sequences including the assembled MAT loci have been deposited in GenBank under the accession numbers listed in the Table. *MAT locus distributed over multiple scaffolds which could not be combined.*

#	CBS number	DTO number	Country of origin	Isolated from	Accession number
1	CBS 554.65	-	Connecticut, USA	Tannin-gallic acid fermentation	PRJNA715116
2	CBS 113.50	DTO 008-C3	Unknown	Leather	MW809487
3	CBS 112.32	DTO 028-I3	Japan	Unknown	MW809488
4	CBS 124.48	DTO 029-B1	Unknown	Unknown	MW809489
5	CBS 118.52	DTO 058-I1	Unknown	Unknown	Incomplete coverage*
6	CBS 147371	DTO 096-A5	Coffee Research Station, Netrakonda, near Chickmagalur, India	Green coffee bean	MW809493
7	CBS 147320	DTO 096-A7	Australia	Grape	MW809494
8	CBS 147321	DTO 096-A9	Svalbard, Norway	Arctic soil	MW809495
9	CBS 147322	DTO 096-C6	Brazil	Coffee	MW809496
10	CBS 147323	DTO 096-D7	Fabula, Turkey	Raisin	MW809497
11	CBS 147324	DTO 096-E1	Unknown	Unknown	MW809498
12	CBS 147482	DTO 175-I5	Portugal	Surface water	Incomplete coverage*
13	CBS 147344	DTO 293-G7	Thailand	Coffee beans (Robusta)	MW809499
14	CBS 133816	DTO 316-E3	Denmark	Black pepper	MW809500
15	CBS 147345	DTO 316-E4	USA	Unknown	MW809501
16	CBS 147346	DTO 321-E6	The Netherlands	CF patient material	MW809502
17	CBS 147347	DTO 326-A7	The Netherlands	Petridish, soft drink factory	MW809503
18	CBS 769.97	DTO 367-D1	Unknown	Leather	MW809504
19	CBS 115989	DTO 367-D6	Unknown	Unknown	MW809505
20	CBS 147352	DTO 368-I1	Mexico	Air next to bottle blower	MW809506
21	CBS 147353	DTO 368-I6	Italy	Food factory of Sanquinetto	MW809507
22	CBS 115988	DTO 059-C7	Unknown	Unknown	MW809491
23	CBS 630.78	DTO 067-H7	South Pacific Islands	Army equipment	MW809492
24	CBS 131.52	DTO 029-C3	Unknown	Leather	MW809490
25	CBS 147343	DTO 291-B7	Thailand	Coffee bean	MW809508
