# Supplementary material for: Genome sequencing of the neotype strain CBS 554.65 reveals the MAT1–2 locus of Aspergillus niger
Source: BMC Genomics. 2021 Sep 21;22:679. doi: 10.1186/s12864-021-07990-8 (PMC8454179; doi:10.1186/s12864-021-07990-8)
Supplement: Supplementary file 2 — Additional file 2: Table S2. List of primers used in this study. [file 12864_2021_7990_MOESM2_ESM.pdf]

Table S2. List of primers used in this study.

Name	Sequence	Purpose
chr5_left_fwd	ACTTATCCCTCGTCAATGA	Check sequencing and assembly CBS 554.65
chr5_left_rev	GGTCGACTTTTTGGGGAAA	Check sequencing and assembly CBS 554.65
chr5_right_fwd_1	TTCTCCATATTGTCAGCCAT	Check sequencing and assembly CBS 554.65
chr5_right_rev_1	CATCGCTTCTTTTCCTCGGA	Check sequencing and assembly CBS 554.65
B150	GTGATCTTAACTTGCCGGG	Check MAT locus orientation
B151	TTCGTTCAACCTCCTTGCC	Check MAT locus orientation
B152	GGTCCGCTAATGAAACAAGAA	Check MAT locus orientation
mat1_1_2F	CCAGACCAATCCTCGCGCAGAC	Fill gaps between genomic scaffolds within the MAT loci of *A. niger* isolates.
mat1_1_1R	AGGTTGTTCTTGGAGCAGCGGC	Fill gaps between genomic scaffolds within the MAT loci of *A. niger* isolates.
mat1_2_2F	TTGGAAGGTGACCTGCGTGTGC	Fill gaps between genomic scaffolds within the MAT loci of *A. niger* isolates.
mat1_2_1R	AGCTAAGGTTGGCCTCGCCTGA	Fill gaps between genomic scaffolds within the MAT loci of *A. niger* isolates.
mat1_2_4F	TCTGGAACGAGGTTGGGGTACA	Fill gaps between genomic scaffolds within the MAT loci of *A. niger* isolates.
mat1_2_3R	GGAAAACAGGGCTTCCCGTCCG	Fill gaps between genomic scaffolds within the MAT loci of *A. niger* isolates.
