# Supplementary material for: Genome sequencing of the neotype strain CBS 554.65 reveals the MAT1–2 locus of Aspergillus niger
Source: BMC Genomics. 2021 Sep 21;22:679. doi: 10.1186/s12864-021-07990-8 (PMC8454179; doi:10.1186/s12864-021-07990-8)
Supplement: Supplementary file 3 — Additional file 3: Table S3. Genome characteristics and found Benchmarking Universal Single-Copy Orthologues (BUSCO) genes of the assembled Aspergillus niger strain CBS 554.65. Table S4. Masked repetitive elements found with RepeatMasker v4.0.9 and tRNA genes found by tRNAscan-SE v1.3.1. [file 12864_2021_7990_MOESM3_ESM.pdf]

Table S3. Genome characteristics and found Benchmarking Universal Single-Copy Orthologues (BUSCO) genes of the assembled Aspergillus niger strain CBS 554.65.

Genome	*A. niger* CBS 554.65
Assembly size (bp)	40,425,233
G+C content (%)	49.57
Scaffolds (>= 0 bp)	17
Scaffolds (>= 1000 bp)	17
Largest scaffold (bp)	6,394,477
N50 (bp)	4,067,305
L50 (scaffolds)	4
N's per 100 kbp	0.00
Complete BUSCO (%)	100.00
Partial BUSCO (%)	0.00

*Table S4. Masked repetitive elements found with RepeatMasker v4.0.9 and tRNA genes found by tRNAscan-SE v1.3.1. *Most repeats that were fragmented by insertions or deletions have been counted as one element.*

Masked element	Number of elements*	Length occupied in bp	Percentage of sequence
SINEs	16	1,147	-
LINEs	127	8,842	0.02%
LTR elements	4	281	-
DNA elements	52	3,841	0.01%
Small RNA	338	163,900	0.41%
Simple repeats	9,791	401,723	0.99%
Low complexity	1,694	90,392	0.22%
tRNA	277	23,237	0.06%
