# Supplementary material for: Genome sequencing of the neotype strain CBS 554.65 reveals the MAT1–2 locus of Aspergillus niger
Source: BMC Genomics. 2021 Sep 21;22:679. doi: 10.1186/s12864-021-07990-8 (PMC8454179; doi:10.1186/s12864-021-07990-8)
Supplement: Supplementary file 4 — Additional file 4: Fig. S1. Assembly of the genome sequence of CBS 554.65 consisting of 17 contigs (in scale). For each contig (black horizontal lines) the annotated ORFs (first row), the GC content (second row) and the conservation compared to CBS 513.88 (third row) are schematically represented. [file 12864_2021_7990_MOESM4_ESM.pdf]

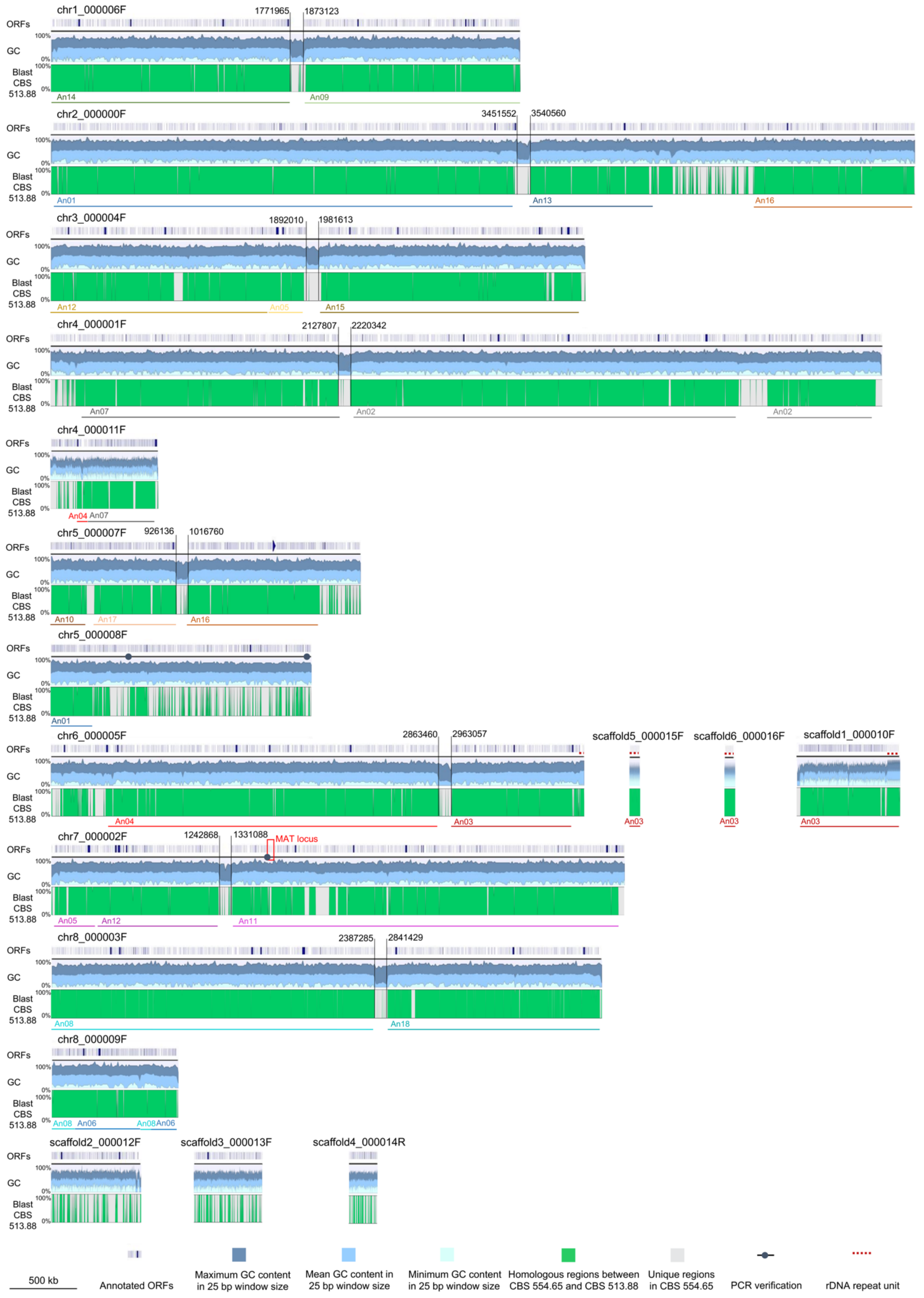


*Figure S1. Assembly of the genome sequence of CBS 554.65 consisting of 17 contigs (in scale). For each contig (black horizontal lines) the annotated ORFs (first row), the GC content (second row) and the conservation compared to CBS 513.88 (third row) are schematically represented. The annotation was obtained with Augustus. The GC content was calculated using a window*

*size of 25 bp. The upper and darker graph represents the maximum GC content value observed in that region, the middle graph represents the mean GC value and the lower graph represents the minimum GC value. The conservation graph (last row) was obtained by blasting each contig of CBS 554.65 against the whole genome of strain CBS 513.88. Green areas indicate genomic regions conserved between the two strains, grey areas indicate regions only found in CBS 554.65 and not in CBS 513.88. Below the conservation graph lines representing the chromosomes of strain CBS 513.88 are reported, as a result of the blast analysis. Chr6_00005F, scaffold1_000010F, scaffold5_000015F and scaffold6_000016F contain the highly repetitive ribosomal DNA (rDNA) gene unit, indicated with a dashed line on top of the scaffolds. Notably, for each of the 8 identified chromosomes, a centromeric region of at least 80 kb could be identified where ORFs are not annotated (indicated with two parallel and vertical lines; the first and the last nucleotide after and before the annotated ORFs, respectively, are indicated). These regions correspond to a decrease in the GC content (as indicated in the GC graph) and are only partially present in the genome of strain CBS 513.88 (grey areas in the blast graph). Dots on chr5_000008F and on chr7_000002F indicate the region where the PCRs were performed. The MAT locus is indicated by a red box on chromosome 7. Additional information on the length of the contigs and the coordinates of the alignments are reported in Table S8 of Additional file 7.*
