# Supplementary material for: Genome sequencing of the neotype strain CBS 554.65 reveals the MAT1–2 locus of Aspergillus niger
Source: BMC Genomics. 2021 Sep 21;22:679. doi: 10.1186/s12864-021-07990-8 (PMC8454179; doi:10.1186/s12864-021-07990-8)
Supplement: Supplementary file 5 — Additional file 5: Fig. S2. Coverage plots of the scaffolds obtained by remapping the reads to the CBS 554.65 genome assembly. [file 12864_2021_7990_MOESM5_ESM.pdf]

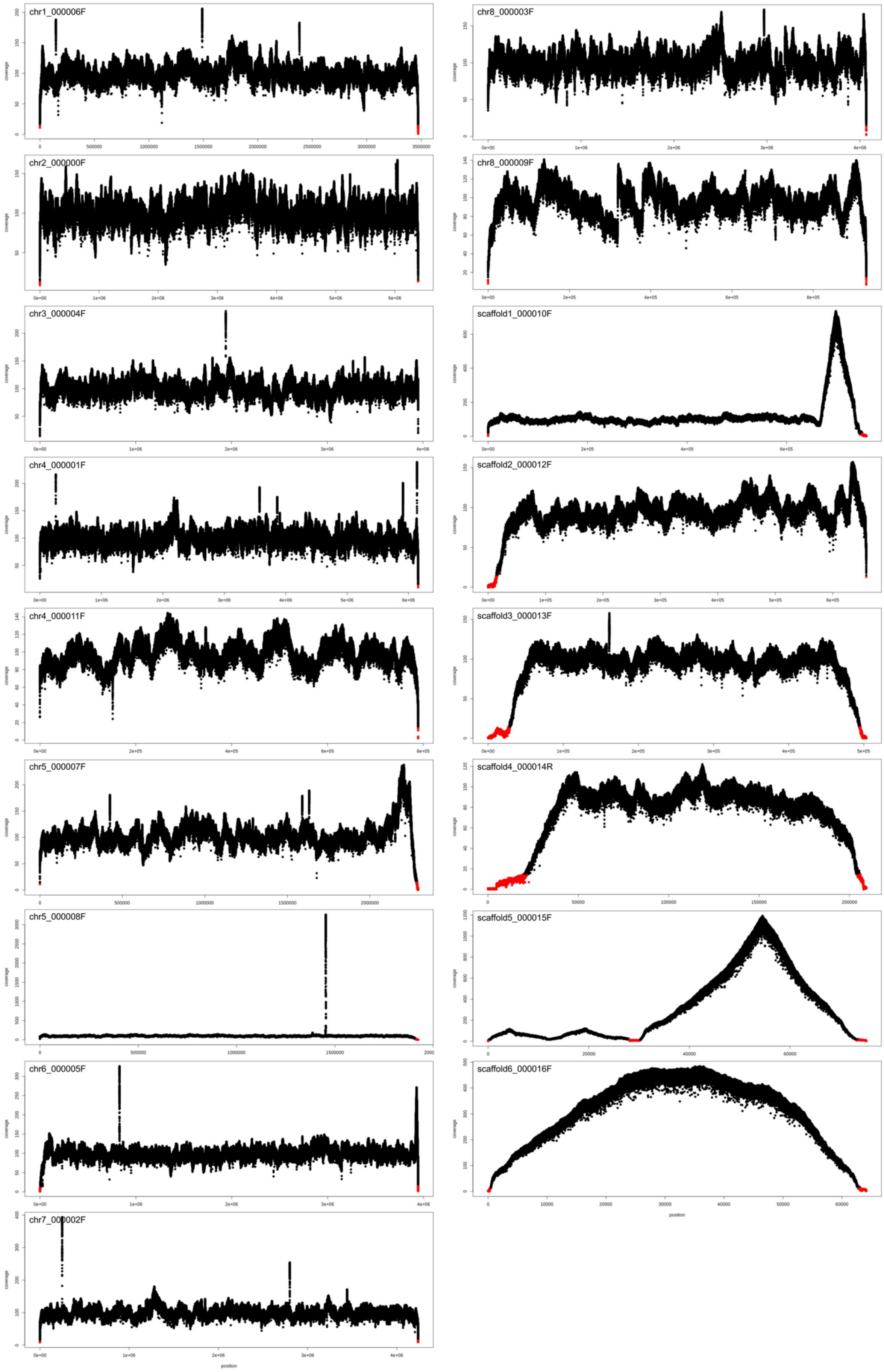

Figure S2. Coverage plots of the scaffolds obtained by remapping the reads to the CBS 554.65 genome assembly.
