# Supplementary material for: Genome sequencing of the neotype strain CBS 554.65 reveals the MAT1–2 locus of Aspergillus niger
Source: BMC Genomics. 2021 Sep 21;22:679. doi: 10.1186/s12864-021-07990-8 (PMC8454179; doi:10.1186/s12864-021-07990-8)
Supplement: Supplementary file 6 — Additional file 6: Table S5. GO term enrichment analysis of the unique GO term set of CBS 554.65 referenced to the entire GO term set of CBS 554.65. The unique CBS 554.65 proteins compared to NRRL3 are 694, of which 176 had at least one GO term assigned. Fig. S3. GO term enrichment analysis of the unique GO term set of CBS 554.65 referenced to the entire GO term set of CBS 554.65, assigned to the biological process ontology. Fig. S4. GO term enrichment analysis of the unique GO term set of CBS 554.65 referenced to the entire GO term set of CBS 554.65, assigned to the molecular function ontology. Table S6. Unique protein sequences in the proteome of CBS 554.65 compared to NRRL3 by a blastp analysis. Table S7. Unique protein sequences in the proteome of NRRL3 compared to the entire proteome of CBS 554.65 by a blastp analysis. [file 12864_2021_7990_MOESM6_ESM.pdf]

Table S5. GO term enrichment analysis of the unique GO term set of CBS 554.65 referenced to the entire GO term set of CBS 554.65. The unique CBS 554.65 proteins compared to NRRL3 are 694, of which 176 had at least one GO term assigned.

GO Term	FDR	GO Term Name	Ontology
GO:0042357	8.8e-08	thiamine diphosphate metabolic process	BP
GO:0006772	1.1e-06	thiamine metabolic process	BP
GO:0006695	1.1e-06	cholesterol biosynthetic process	BP
GO:0042723	1.1e-06	thiamine-containing compound metabolic process	BP
GO:0008203	2.5e-06	cholesterol metabolic process	BP
GO:0090502	3.5e-06	RNA phosphodiester bond hydrolysis endonucleolytic	BP
GO:0090501	3.1e-05	RNA phosphodiester bond hydrolysis	BP
GO:0072527	0.0007	pyrimidine-containing compound metabolic process	BP
GO:0090305	0.0019	nucleic acid phosphodiester bond hydrolysis	BP
GO:0009116	0.0028	nucleoside metabolic process	BP
GO:1901657	0.0036	glycosyl compound metabolic process	BP
GO:1902653	0.0044	secondary alcohol biosynthetic process	BP
GO:0030162	0.0047	regulation of proteolysis	BP
GO:1902652	0.006	secondary alcohol metabolic process	BP
GO:0016126	0.007	sterol biosynthetic process	BP
GO:0006767	0.008	water-soluble vitamin metabolic process	BP
GO:0006766	0.008	vitamin metabolic process	BP
GO:0006278	0.008	RNA-dependent DNA biosynthetic process	BP
GO:0046165	0.017	alcohol biosynthetic process	BP
GO:0006694	0.023	steroid biosynthetic process	BP
GO:0016125	0.043	sterol metabolic process	BP
GO:0050333	3.7e-10	thiamin-triphosphatase activity	MF
GO:0004525	8.2e-10	ribonuclease III activity	MF
GO:0032296	8.2e-10	double-stranded RNA-specific ribonuclease activity	MF
GO:0046983	2.2e-08	protein dimerization activity	MF
GO:0016891	4.7e-08	endoribonuclease activity	MF
GO:0016893	1.3e-07	endonuclease activity	MF
GO:0004521	1.8e-07	endoribonuclease activity	MF
GO:0004631	2.6e-07	phosphomevalonate kinase activity	MF
GO:0004540	1.4e-06	ribonuclease activity	MF
GO:0031625	3.7e-05	ubiquitin protein ligase binding	MF
GO:0016776	3.7e-05	phosphotransferase activity	MF
GO:0004519	3.7e-05	endonuclease activity	MF
GO:0044389	4.3e-05	ubiquitin-like protein ligase binding	MF
GO:0004518	0.00015	nuclease activity	MF
GO:0003964	0.0026	RNA-directed DNA polymerase activity	MF
GO:0005515	0.0056	protein binding	MF
GO:0019899	0.0091	enzyme binding	MF
GO:0034061	0.02	DNA polymerase activity	MF

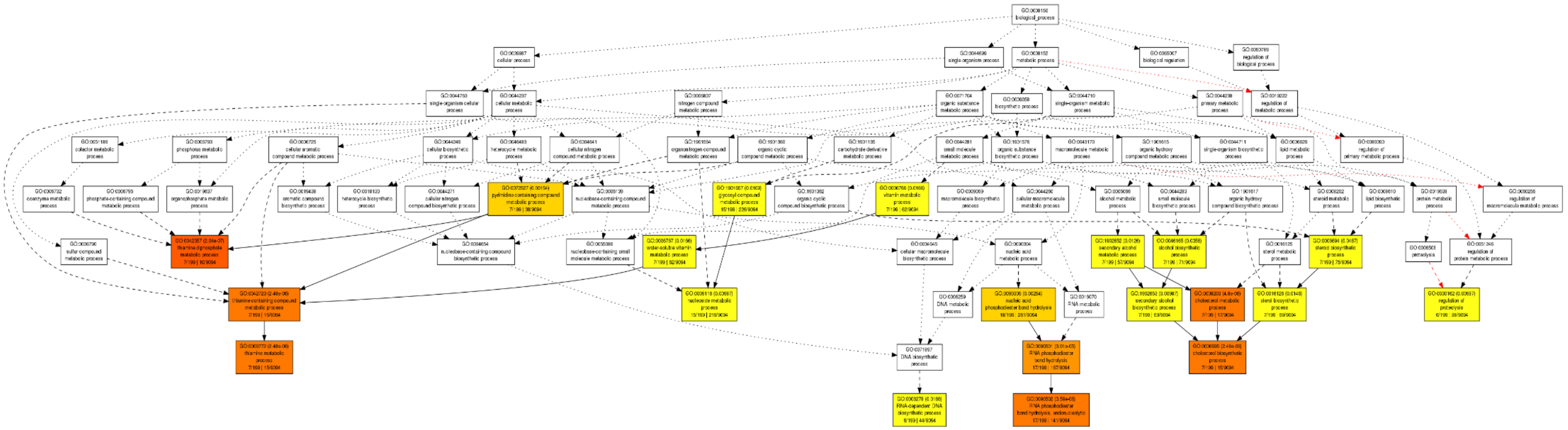

Figure S3. GO term enrichment analysis of the unique GO term set of CBS 554.65 referenced to the entire GO term set of CBS 554.65, assigned to the biological process ontology.

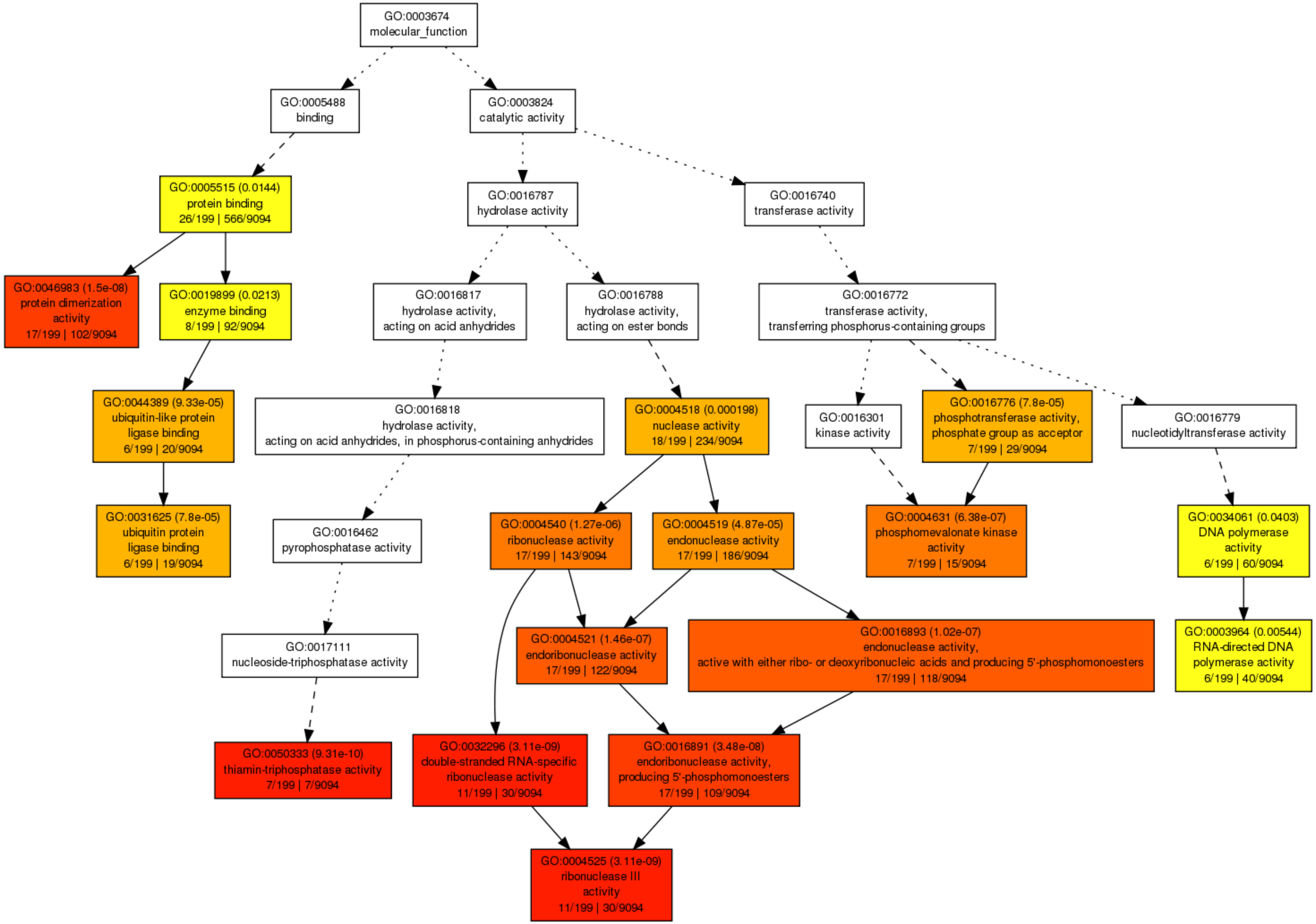

Figure S4. *GO term enrichment analysis of the unique GO term set of CBS 554.65 referenced to the entire GO term set of CBS 554.65, assigned to the molecular function ontology.*

Table S6. Unique protein sequences in the proteome of CBS 554.65 compared to NRRL3 by a blastp analysis.

	Query	Number of HSPs	Lowest E-value	Accession (E-value)	Greatest identity %	Greatest positive %	Greatest HSP length	Greatest bit score
1	chr1_000006F CDS g9867	0	no hit	not available	-	-	-	-
2	chr1_000006F CDS g10456	0	no hit	not available	-	-	-	-
3	chr1_000006F CDS g10971	0	no hit	not available	-	-	-	-
4	chr2_000000F CDS g3842	0	no hit	not available	-	-	-	-
5	chr2_000000F CDS g5306	0	no hit	not available	-	-	-	-
6	chr2_000000F CDS g5844	0	no hit	not available	-	-	-	-
7	chr2_000000F CDS g5465	0	no hit	not available	-	-	-	-
8	chr2_000000F CDS g5422	0	no hit	not available	-	-	-	-
9	chr2_000000F CDS g5362	0	no hit	not available	-	-	-	-
10	chr2_000000F CDS g5341	0	no hit	not available	-	-	-	-
11	chr2_000000F CDS g5316	0	no hit	not available	-	-	-	-
12	chr2_000000F CDS g5425	0	no hit	not available	-	-	-	-
13	chr2_000000F CDS g5310	0	no hit	not available	-	-	-	-
14	chr2_000000F CDS g5340	0	no hit	not available	-	-	-	-
15	chr2_000000F CDS g5331	0	no hit	not available	-	-	-	-
16	chr2_000000F CDS g5426	0	no hit	not available	-	-	-	-
17	chr2_000000F CDS g5359	0	no hit	not available	-	-	-	-
18	chr2_000000F CDS g5438	0	no hit	not available	-	-	-	-
19	chr2_000000F CDS g4893	0	no hit	not available	-	-	-	-
20	chr2_000000F CDS g5375	0	no hit	not available	-	-	-	-
21	chr2_000000F CDS g5843	0	no hit	not available	-	-	-	-
22	chr2_000000F CDS g5424	0	no hit	not available	-	-	-	-
23	chr2_000000F CDS g5402	0	no hit	not available	-	-	-	-
24	chr2_000000F CDS g5406	0	no hit	not available	-	-	-	-
25	chr2_000000F CDS g5364	0	no hit	not available	-	-	-	-
26	chr2_000000F CDS g5311	0	no hit	not available	-	-	-	-
27	chr2_000000F CDS g5439	0	no hit	not available	-	-	-	-
28	chr2_000000F CDS g5373	0	no hit	not available	-	-	-	-
29	chr2_000000F CDS g5323	0	no hit	not available	-	-	-	-
30	chr2_000000F CDS g4888	0	no hit	not available	-	-	-	-
31	chr2_000000F CDS g5397	0	no hit	not available	-	-	-	-
32	chr2_000000F CDS g5411	0	no hit	not available	-	-	-	-
33	chr2_000000F CDS g5342	0	no hit	not available	-	-	-	-
34	chr2_000000F CDS g5440	0	no hit	not available	-	-	-	-
35	chr2_000000F CDS g5098	0	no hit	not available	-	-	-	-

36	chr2_000000F CDS g5419	0	no hit	not available	-	-	-	-
37	chr2_000000F CDS g5394	0	no hit	not available	-	-	-	-
38	chr2_000000F CDS g5332	0	no hit	not available	-	-	-	-
39	chr2_000000F CDS g5321	0	no hit	not available	-	-	-	-
40	chr2_000000F CDS g4894	0	no hit	not available	-	-	-	-
41	chr2_000000F CDS g5654	0	no hit	not available	-	-	-	-
42	chr2_000000F CDS g5393	0	no hit	not available	-	-	-	-
43	chr2_000000F CDS g5395	0	no hit	not available	-	-	-	-
44	chr3_000004F CDS g7272	0	no hit	not available	-	-	-	-
45	chr3_000004F CDS g7274	0	no hit	not available	-	-	-	-
46	chr3_000004F CDS g7281	0	no hit	not available	-	-	-	-
47	chr4_000001F CDS g892	0	no hit	not available	-	-	-	-
48	chr4_000001F CDS g167	0	no hit	not available	-	-	-	-
49	chr4_000001F CDS g134	0	no hit	not available	-	-	-	-
50	chr4_000001F CDS g158	0	no hit	not available	-	-	-	-
51	chr4_000001F CDS g189	0	no hit	not available	-	-	-	-
52	chr4_000001F CDS g159	0	no hit	not available	-	-	-	-
53	chr4_000001F CDS g151	0	no hit	not available	-	-	-	-
54	chr4_000001F CDS g138	0	no hit	not available	-	-	-	-
55	chr4_000001F CDS g160	0	no hit	not available	-	-	-	-
56	chr4_000001F CDS g1707	0	no hit	not available	-	-	-	-
57	chr4_000001F CDS g1662	0	no hit	not available	-	-	-	-
58	chr4_000001F CDS g1697	0	no hit	not available	-	-	-	-
59	chr4_000001F CDS g1663	0	no hit	not available	-	-	-	-
60	chr4_000001F CDS g1664	0	no hit	not available	-	-	-	-
61	chr4_000001F CDS g1668	0	no hit	not available	-	-	-	-
62	chr4_000001F CDS g1710	0	no hit	not available	-	-	-	-
63	chr4_000001F CDS g1677	0	no hit	not available	-	-	-	-
64	chr4_000001F CDS g1669	0	no hit	not available	-	-	-	-
65	chr4_000001F CDS g1678	0	no hit	not available	-	-	-	-
66	chr4_000001F CDS g1708	0	no hit	not available	-	-	-	-
67	chr4_000001F CDS g1711	0	no hit	not available	-	-	-	-
68	chr4_000001F CDS g1675	0	no hit	not available	-	-	-	-
69	chr4_000011F CDS g2236	0	no hit	not available	-	-	-	-
70	chr4_000011F CDS g2268	0	no hit	not available	-	-	-	-
71	chr4_000011F CDS g2270	0	no hit	not available	-	-	-	-
72	chr4_000011F CDS g2227	0	no hit	not available	-	-	-	-
73	chr4_000011F CDS g2234	0	no hit	not available	-	-	-	-

74	chr4_000011F CDS g2261	0	no hit	not available	-	-	-	-
75	chr4_000011F CDS g2220	0	no hit	not available	-	-	-	-
76	chr4_000011F CDS g2235	0	no hit	not available	-	-	-	-
77	chr5_000007F CDS g8490	0	no hit	not available	-	-	-	-
78	chr5_000007F CDS g8545	0	no hit	not available	-	-	-	-
79	chr5_000007F CDS g7919	0	no hit	not available	-	-	-	-
80	chr5_000007F CDS g8381	0	no hit	not available	-	-	-	-
81	chr5_000007F CDS g7898	0	no hit	not available	-	-	-	-
82	chr5_000007F CDS g8514	0	no hit	not available	-	-	-	-
83	chr5_000007F CDS g8531	0	no hit	not available	-	-	-	-
84	chr5_000007F CDS g8486	0	no hit	not available	-	-	-	-
85	chr5_000007F CDS g8503	0	no hit	not available	-	-	-	-
86	chr5_000007F CDS g7900	0	no hit	not available	-	-	-	-
87	chr5_000007F CDS g8547	0	no hit	not available	-	-	-	-
88	chr5_000008F CDS g7793	0	no hit	not available	-	-	-	-
89	chr5_000008F CDS g7488	0	no hit	not available	-	-	-	-
90	chr5_000008F CDS g7532	0	no hit	not available	-	-	-	-
91	chr5_000008F CDS g7779	0	no hit	not available	-	-	-	-
92	chr5_000008F CDS g7519	0	no hit	not available	-	-	-	-
93	chr5_000008F CDS g7487	0	no hit	not available	-	-	-	-
94	chr5_000008F CDS g7812	0	no hit	not available	-	-	-	-
95	chr5_000008F CDS g7512	0	no hit	not available	-	-	-	-
96	chr5_000008F CDS g7816	0	no hit	not available	-	-	-	-
97	chr5_000008F CDS g7810	0	no hit	not available	-	-	-	-
98	chr5_000008F CDS g7688	0	no hit	not available	-	-	-	-
99	chr5_000008F CDS g7497	0	no hit	not available	-	-	-	-
100	chr5_000008F CDS g7601	0	no hit	not available	-	-	-	-
101	chr5_000008F CDS g7691	0	no hit	not available	-	-	-	-
102	chr5_000008F CDS g7822	0	no hit	not available	-	-	-	-
103	chr5_000008F CDS g7516	0	no hit	not available	-	-	-	-
104	chr5_000008F CDS g7680	0	no hit	not available	-	-	-	-
105	chr5_000008F CDS g7498	0	no hit	not available	-	-	-	-
106	chr5_000008F CDS g7503	0	no hit	not available	-	-	-	-
107	chr5_000008F CDS g7602	0	no hit	not available	-	-	-	-
108	chr5_000008F CDS g7751	0	no hit	not available	-	-	-	-
109	chr5_000008F CDS g7574	0	no hit	not available	-	-	-	-
110	chr5_000008F CDS g7882	0	no hit	not available	-	-	-	-
111	chr5_000008F CDS g7531	0	no hit	not available	-	-	-	-

112	chr5_000008F CDS g7483	0	no hit	not available	-	-	-	-
113	chr5_000008F CDS g7499	0	no hit	not available	-	-	-	-
114	chr5_000008F CDS g7552	0	no hit	not available	-	-	-	-
115	chr5_000008F CDS g7544	0	no hit	not available	-	-	-	-
116	chr5_000008F CDS g7562	0	no hit	not available	-	-	-	-
117	chr5_000008F CDS g7478	0	no hit	not available	-	-	-	-
118	chr5_000008F CDS g7639	0	no hit	not available	-	-	-	-
119	chr5_000008F CDS g7729	0	no hit	not available	-	-	-	-
120	chr5_000008F CDS g7508	0	no hit	not available	-	-	-	-
121	chr5_000008F CDS g7575	0	no hit	not available	-	-	-	-
122	chr5_000008F CDS g7873	0	no hit	not available	-	-	-	-
123	chr5_000008F CDS g7814	0	no hit	not available	-	-	-	-
124	chr5_000008F CDS g7760	0	no hit	not available	-	-	-	-
125	chr5_000008F CDS g7557	0	no hit	not available	-	-	-	-
126	chr5_000008F CDS g7502	0	no hit	not available	-	-	-	-
127	chr5_000008F CDS g7682	0	no hit	not available	-	-	-	-
128	chr5_000008F CDS g7730	0	no hit	not available	-	-	-	-
129	chr5_000008F CDS g7664	0	no hit	not available	-	-	-	-
130	chr5_000008F CDS g7831	0	no hit	not available	-	-	-	-
131	chr5_000008F CDS g7743	0	no hit	not available	-	-	-	-
132	chr5_000008F CDS g7600	0	no hit	not available	-	-	-	-
133	chr5_000008F CDS g7564	0	no hit	not available	-	-	-	-
134	chr5_000008F CDS g7681	0	no hit	not available	-	-	-	-
135	chr5_000008F CDS g7567	0	no hit	not available	-	-	-	-
136	chr5_000008F CDS g7566	0	no hit	not available	-	-	-	-
137	chr5_000008F CDS g7701	0	no hit	not available	-	-	-	-
138	chr5_000008F CDS g7496	0	no hit	not available	-	-	-	-
139	chr5_000008F CDS g7843	0	no hit	not available	-	-	-	-
140	chr5_000008F CDS g7683	0	no hit	not available	-	-	-	-
141	chr5_000008F CDS g7886	0	no hit	not available	-	-	-	-
142	chr5_000008F CDS g7555	0	no hit	not available	-	-	-	-
143	chr5_000008F CDS g7626	0	no hit	not available	-	-	-	-
144	chr5_000008F CDS g7826	0	no hit	not available	-	-	-	-
145	chr5_000008F CDS g7728	0	no hit	not available	-	-	-	-
146	chr5_000008F CDS g7795	0	no hit	not available	-	-	-	-
147	chr5_000008F CDS g7830	0	no hit	not available	-	-	-	-
148	chr5_000008F CDS g7511	0	no hit	not available	-	-	-	-
149	chr5_000008F CDS g7479	0	no hit	not available	-	-	-	-

150	chr5_000008F CDS g7568	0	no hit	not available	-	-	-	-
151	chr5_000008F CDS g7543	0	no hit	not available	-	-	-	-
152	chr5_000008F CDS g7526	0	no hit	not available	-	-	-	-
153	chr5_000008F CDS g7891	0	no hit	not available	-	-	-	-
154	chr5_000008F CDS g7534	0	no hit	not available	-	-	-	-
155	chr5_000008F CDS g7494	0	no hit	not available	-	-	-	-
156	chr5_000008F CDS g7752	0	no hit	not available	-	-	-	-
157	chr5_000008F CDS g7580	0	no hit	not available	-	-	-	-
158	chr5_000008F CDS g7612	0	no hit	not available	-	-	-	-
159	chr5_000008F CDS g7889	0	no hit	not available	-	-	-	-
160	chr5_000008F CDS g7482	0	no hit	not available	-	-	-	-
161	chr5_000008F CDS g7690	0	no hit	not available	-	-	-	-
162	chr5_000008F CDS g7737	0	no hit	not available	-	-	-	-
163	chr5_000008F CDS g7710	0	no hit	not available	-	-	-	-
164	chr5_000008F CDS g7813	0	no hit	not available	-	-	-	-
165	chr5_000008F CDS g7533	0	no hit	not available	-	-	-	-
166	chr5_000008F CDS g7715	0	no hit	not available	-	-	-	-
167	chr5_000008F CDS g7551	0	no hit	not available	-	-	-	-
168	chr5_000008F CDS g7692	0	no hit	not available	-	-	-	-
169	chr6_000005F CDS g2774	0	no hit	not available	-	-	-	-
170	chr6_000005F CDS g2797	0	no hit	not available	-	-	-	-
171	chr6_000005F CDS g2759	0	no hit	not available	-	-	-	-
172	chr6_000005F CDS g2753	0	no hit	not available	-	-	-	-
173	chr6_000005F CDS g2731	0	no hit	not available	-	-	-	-
174	chr6_000005F CDS g2754	0	no hit	not available	-	-	-	-
175	chr6_000005F CDS g2767	0	no hit	not available	-	-	-	-
176	chr6_000005F CDS g3180	0	no hit	not available	-	-	-	-
177	chr6_000005F CDS g2734	0	no hit	not available	-	-	-	-
178	chr6_000005F CDS g2738	0	no hit	not available	-	-	-	-
179	chr6_000005F CDS g2751	0	no hit	not available	-	-	-	-
180	chr6_000005F CDS g2744	0	no hit	not available	-	-	-	-
181	chr6_000005F CDS g2760	0	no hit	not available	-	-	-	-
182	chr6_000005F CDS g2739	0	no hit	not available	-	-	-	-
183	chr6_000005F CDS g2710	0	no hit	not available	-	-	-	-
184	chr6_000005F CDS g2749	0	no hit	not available	-	-	-	-
185	chr6_000005F CDS g2726	0	no hit	not available	-	-	-	-
186	chr7_000002F CDS g9161	0	no hit	not available	-	-	-	-
187	chr7_000002F CDS g9162	0	no hit	not available	-	-	-	-

188	chr7_000002F CDS g9160	0	no hit	not available	-	-	-	-
189	chr7_000002F CDS g9164	0	no hit	not available	-	-	-	-
190	chr7_000002F CDS g9165	0	no hit	not available	-	-	-	-
191	chr7_000002F CDS g9225	0	no hit	not available	-	-	-	-
192	chr7_000002F CDS g9542	0	no hit	not available	-	-	-	-
193	chr7_000002F CDS g9406	0	no hit	not available	-	-	-	-
194	chr7_000002F CDS g9224	0	no hit	not available	-	-	-	-
195	chr7_000002F CDS g9222	0	no hit	not available	-	-	-	-
196	chr7_000002F CDS g9405	0	no hit	not available	-	-	-	-
197	chr8_000009F CDS g6043	0	no hit	not available	-	-	-	-
198	scaffold1_000010F CDS g2205	0	no hit	not available	-	-	-	-
199	scaffold1_000010F CDS g2194	0	no hit	not available	-	-	-	-
200	scaffold2_000012F CDS g2584	0	no hit	not available	-	-	-	-
201	scaffold2_000012F CDS g2612	0	no hit	not available	-	-	-	-
202	scaffold2_000012F CDS g2543	0	no hit	not available	-	-	-	-
203	scaffold2_000012F CDS g2589	0	no hit	not available	-	-	-	-
204	scaffold2_000012F CDS g2482	0	no hit	not available	-	-	-	-
205	scaffold2_000012F CDS g2551	0	no hit	not available	-	-	-	-
206	scaffold2_000012F CDS g2507	0	no hit	not available	-	-	-	-
207	scaffold2_000012F CDS g2542	0	no hit	not available	-	-	-	-
208	scaffold2_000012F CDS g2586	0	no hit	not available	-	-	-	-
209	scaffold2_000012F CDS g2474	0	no hit	not available	-	-	-	-
210	scaffold2_000012F CDS g2541	0	no hit	not available	-	-	-	-
211	scaffold2_000012F CDS g2624	0	no hit	not available	-	-	-	-
212	scaffold2_000012F CDS g2535	0	no hit	not available	-	-	-	-
213	scaffold2_000012F CDS g2634	0	no hit	not available	-	-	-	-
214	scaffold2_000012F CDS g2478	0	no hit	not available	-	-	-	-
215	scaffold2_000012F CDS g2471	0	no hit	not available	-	-	-	-
216	scaffold2_000012F CDS g2562	0	no hit	not available	-	-	-	-
217	scaffold2_000012F CDS g2633	0	no hit	not available	-	-	-	-
218	scaffold2_000012F CDS g2510	0	no hit	not available	-	-	-	-
219	scaffold3_000013F CDS g127	0	no hit	not available	-	-	-	-
220	scaffold3_000013F CDS g125	0	no hit	not available	-	-	-	-
221	scaffold3_000013F CDS g104	0	no hit	not available	-	-	-	-
222	scaffold3_000013F CDS g6	0	no hit	not available	-	-	-	-
223	scaffold3_000013F CDS g78	0	no hit	not available	-	-	-	-
224	scaffold3_000013F CDS g45	0	no hit	not available	-	-	-	-
225	scaffold3_000013F CDS g89	0	no hit	not available	-	-	-	-

226	scaffold3_000013F CDS g58	0	no hit	not available	-	-	-	-
227	scaffold3_000013F CDS g37	0	no hit	not available	-	-	-	-
228	scaffold3_000013F CDS g10	0	no hit	not available	-	-	-	-
229	scaffold3_000013F CDS g26	0	no hit	not available	-	-	-	-
230	scaffold3_000013F CDS g44	0	no hit	not available	-	-	-	-
231	scaffold3_000013F CDS g8	0	no hit	not available	-	-	-	-
232	scaffold3_000013F CDS g3	0	no hit	not available	-	-	-	-
233	scaffold4_000014R CDS g2692	0	no hit	not available	-	-	-	-
234	scaffold4_000014R CDS g2639	0	no hit	not available	-	-	-	-
235	scaffold4_000014R CDS g2645	0	no hit	not available	-	-	-	-
236	scaffold4_000014R CDS g2647	0	no hit	not available	-	-	-	-
237	scaffold4_000014R CDS g2690	0	no hit	not available	-	-	-	-
238	chr1_000006F CDS g9875	2	1.99E-42	chr_701_(+1)	39.68	56.75	252	154.84
239	chr1_000006F CDS g10229	2	1.15E-92	chr_102_(-2)	75.61	78.05	207	297.36
240	chr1_000006F CDS g10831	2	3.52E-27	chr_202_CDS_jgi.p_Aspni_NRRL3_1_2757	29.45	45.31	292	111.69
241	chr1_000006F CDS g10739	1	1.14E-68	chr_101_(+3)	84.56	84.56	136	224.94
242	chr1_000006F CDS g10969	2	1.52E-36	chr_101_(+2)	85.07	89.74	78	134.42
243	chr2_000000F CDS g4033	2	8.55E-09	chr_502_CDS_jgi.p_Aspni_NRRL3_1_6990	35.25	49.64	135	58.54
244	chr2_000000F CDS g4502	2	4.90E-48	chr_202_(-3)	72.8	72.8	125	165.62
245	chr2_000000F CDS g5369	2	4.62E-24	chr_601_(-2)	51.38	63.3	130	100.52
246	chr2_000000F CDS g5382	2	5.88E-128	chr_601_(+1)	68.45	76.79	385	416
247	chr2_000000F CDS g4886	2	4.28E-44	chr_201_(+2)	38.86	55.43	312	175.64
248	chr2_000000F CDS g5392	1	4.09E-03	chr_601_CDS_jgi.p_Aspni_NRRL3_1_8719	25.18	47.48	139	40.43
249	chr2_000000F CDS g5344	1	4.50E-03	chr_601_CDS_jgi.p_Aspni_NRRL3_1_8719	25.18	47.48	139	40.43
250	chr2_000000F CDS g5281	2	2.55E-42	chr_401_CDS_jgi.p_Aspni_NRRL3_1_4334	30.02	47.89	375	161.77
251	chr2_000000F CDS g4975	1	4.05E-37	chr_201_(+2)	66.35	66.35	104	132.49
252	chr2_000000F CDS g5387	3	7.73E-130	chr_601_(-1)	85.71	90.16	244	420.62
253	chr2_000000F CDS g5368	2	2.86E-65	chr_601_(+3)	38.66	56.51	497	231.11
254	chr2_000000F CDS g5322	2	1.63E-52	chr_601_(+1)	34.67	54.67	511	193.74
255	chr2_000000F CDS g5350	2	3.51E-26	chr_601_(+1)	55.17	68.28	258	109.77
256	chr2_000000F CDS g5365	2	1.41E-22	chr_601_(+1)	42.25	66.2	142	99.75
257	chr2_000000F CDS g5414	1	2.72E-135	chr_601_(+1)	68.34	80.25	319	426.02
258	chr2_000000F CDS g5371	3	1.54E-63	chr_601_(+3)	86.63	89.84	183	223.02
259	chr2_000000F CDS g4883	2	3.48E-16	chr_102_(+2)	63.29	72.73	78	85.11
260	chr2_000000F CDS g5338	2	6.47E-11	chr_601_(-3)	69.23	79.49	53	63.93
261	chr2_000000F CDS g5374	2	1.90E-14	chr_601_(-1)	82.35	86.27	129	78.57
262	chr2_000000F CDS g5291	2	1.79E-45	chr_601_(-2)	54.59	67.15	207	163.31
263	chr2_000000F CDS g5334	2	4.67E-16	chr_502_(+1)	50.79	71.43	74	81.26

264	chr2_000000F CDS g5435	2	1.12E-18	chr_601_(-3)	84.91	90.57	53	89.35
265	chr2_000000F CDS g4887	2	1.12E-60	chr_502_CDS_jgi.p_Aspni_NRRL3_1_7164	28.04	42.35	867	226.87
266	chr2_000000F CDS g5417	2	3.60E-15	chr_601_(-3)	71.43	80	68	75.87
267	chr2_000000F CDS g4890	1	2.28E-05	chr_502_(-1)	31.2	49.6	123	50.45
268	chr2_000000F CDS g5378	2	3.21E-13	chr_601_(-3)	59.57	74.47	68	70.09
269	chr2_000000F CDS g5381	2	1.12E-109	chr_601_(+1)	68.33	81.67	291	354.37
270	chr2_000000F CDS g5352	3	9.09E-174	chr_601_(+1)	79.69	85.16	506	545.43
271	chr2_000000F CDS g5687	1	7.20E-37	chr_502_(+3)	91.59	92.52	107	134.81
272	chr2_000000F CDS g5328	2	6.43E-09	chr_302_CDS_jgi.p_Aspni_NRRL3_1_3854	46.88	56.25	64	54.3
273	chr2_000000F CDS g5391	2	1.03E-16	chr_101_CDS_jgi.p_Aspni_NRRL3_1_152	27.56	46.67	417	87.04
274	chr2_000000F CDS g5317	3	4.33E-27	chr_601_(-3)	83.67	87.04	66	110.15
275	chr2_000000F CDS g5380	2	1.01E-39	chr_601_(+3)	66.96	72.32	129	143.28
276	chr2_000000F CDS g5420	3	6.20E-16	chr_601_(-1)	88.89	94.44	68	77.8
277	chr2_000000F CDS g5287	2	9.51E-113	chr_601_(-1)	56.23	70.72	345	363.23
278	chr2_000000F CDS g5333	2	5.04E-05	chr_502_CDS_jgi.p_Aspni_NRRL3_1_6646	30.56	44.44	109	45.82
279	chr2_000000F CDS g5430	2	1.33E-20	chr_601_(+1)	79.63	88.89	60	89.35
280	chr2_000000F CDS g5776	2	1.96E-22	chr_502_(-3)	92.31	94.87	60	91.28
281	chr2_000000F CDS g5354	2	1.55E-22	chr_601_(+3)	84.78	93.48	64	95.52
282	chr2_000000F CDS g4892	2	1.71E-11	chr_702_CDS_jgi.p_Aspni_NRRL3_1_9853	25.24	46.15	391	70.86
283	chr2_000000F CDS g5385	2	9.36E-51	chr_202_(+1)	45.13	67.69	195	188.73
284	chr2_000000F CDS g5305	2	2.05E-07	chr_502_(-3)	41.51	61.6	124	57.38
285	chr2_000000F CDS g5288	2	1.08E-05	chr_601_(-2)	40	56.32	87	46.98
286	chr2_000000F CDS g5407	2	5.02E-32	chr_601_(+1)	43.11	66.47	167	124.79
287	chr2_000000F CDS g5312	2	2.58E-05	chr_601_(+2)	26.05	48.74	116	49.29
288	chr2_000000F CDS g5383	1	2.31E-07	chr_601_(+2)	47.54	62.3	61	53.53
289	chr2_000000F CDS g4891	2	8.05E-20	chr_402_CDS_NRRL3_05747	29.73	45.95	200	91.66
290	chr2_000000F CDS g5277	2	3.18E-23	chr_201_CDS_jgi.p_Aspni_NRRL3_1_1389	29.1	48.32	314	100.91
291	chr2_000000F CDS g5345	2	1.03E-16	chr_101_CDS_jgi.p_Aspni_NRRL3_1_152	27.56	46.67	417	87.04
292	chr2_000000F CDS g5408	2	3.08E-60	chr_601_(-2)	54.32	70.37	243	207.22
293	chr2_000000F CDS g5421	2	5.45E-06	chr_402_CDS_NRRL3_05106	26.43	45	131	50.83
294	chr2_000000F CDS g5329	2	1.33E-15	chr_202_CDS_jgi.p_Aspni_NRRL3_1_2020	32.71	47.66	203	80.11
295	chr2_000000F CDS g5326	2	7.54E-10	chr_601_(+1)	34.07	64.84	121	60.46
296	chr2_000000F CDS g5301	2	1.42E-19	chr_601_(+2)	79.63	88.89	59	86.66
297	chr2_000000F CDS g5343	2	2.70E-15	chr_601_CDS_jgi.p_Aspni_NRRL3_1_8719	43.84	65.75	73	68.17
298	chr2_000000F CDS g5379	3	2.88E-73	chr_601_(+1)	58.82	64.71	504	254.22
299	chr2_000000F CDS g5361	2	1.26E-33	chr_702_CDS_jgi.p_Aspni_NRRL3_1_9382	37.58	55.41	261	124.79
300	chr2_000000F CDS g5289	2	1.04E-65	chr_601_(+3)	38.24	56.09	497	232.65
301	chr2_000000F CDS g5396	2	1.07E-58	chr_102_CDS_jgi.p_Aspni_NRRL3_1_822	28.34	47.28	640	211.46

302	chr2_000000F CDS g5437	2	4.37E-60	chr_102_CDS_jgi.p_Aspni_NRRL3_1_822	28.7	47.48	619	221.48
303	chr2_000000F CDS g5307	2	6.14E-10	chr_502_(+1)	43.48	56.52	105	64.7
304	chr2_000000F CDS g5292	2	3.69E-32	chr_601_(+1)	43.11	66.47	167	125.18
305	chr2_000000F CDS g5320	2	1.05E-11	chr_601_(-1)	64.15	75.47	68	65.86
306	chr2_000000F CDS g5388	8	1.75E-07	chr_102_(-2)	25.68	54.73	204	57
307	chr2_000000F CDS g5370	2	2.04E-94	chr_601_(-1)	53.18	67.63	345	310.46
308	chr2_000000F CDS g4884	2	2.40E-03	chr_201_(-1)	44.64	64.29	57	43.9
309	chr2_000000F CDS g5358	2	2.04E-30	chr_402_(-3)	46.15	66.03	156	119.01
310	chr2_000000F CDS g5318	2	1.51E-15	chr_502_CDS_jgi.p_Aspni_NRRL3_1_7176	30.5	48.23	141	73.17
311	chr2_000000F CDS g5339	2	1.23E-42	chr_102_CDS_jgi.p_Aspni_NRRL3_1_822	27.87	45.9	513	163.7
312	chr2_000000F CDS g5068	2	4.38E-25	chr_201_(+2)	85.29	88.24	68	99.37
313	chr3_000004F CDS g6613	2	7.71E-172	chr_301_(-1)	84.28	84.59	318	530.02
314	chr3_000004F CDS g7275	2	5.36E-10	chr_202_(+1)	47.76	61.19	271	61.62
315	chr3_000004F CDS g7270	1	7.31E-39	chr_202_(-3)	86.08	92.41	79	145.59
316	chr3_000004F CDS g7273	2	3.76E-07	chr_301_(+3)	52.78	66.67	65	49.68
317	chr3_000004F CDS g7277	2	1.72E-43	chr_502_CDS_jgi.p_Aspni_NRRL3_1_7156	35.2	54	246	155.61
318	chr4_000001F CDS g200	3	1.84E-39	chr_401_(+2)	86.05	94.19	107	145.21
319	chr4_000001F CDS g142	1	5.41E-12	chr_502_(+2)	54	82	50	64.7
320	chr4_000001F CDS g166	2	4.18E-08	chr_301_CDS_jgi.p_Aspni_NRRL3_1_3222	30.67	42.44	276	55.45
321	chr4_000001F CDS g143	2	4.43E-04	chr_601_(+2)	21.16	42.32	237	45.05
322	chr4_000001F CDS g150	2	3.82E-14	chr_502_(+2)	68.09	76.6	53	68.17
323	chr4_000001F CDS g673	3	3.83E-60	chr_701_(+1)	47.94	66.67	267	213.77
324	chr4_000001F CDS g165	2	1.57E-05	chr_101_CDS_jgi.p_Aspni_NRRL3_1_123	34.48	49.43	107	46.21
325	chr4_000001F CDS g180	2	4.41E-81	chr_601_(-2)	70.12	80.08	241	266.93
326	chr4_000001F CDS g199	1	1.04E-13	chr_401_(+1)	78.05	85.37	41	66.63
327	chr4_000001F CDS g198	2	2.03E-36	chr_401_(-3)	73.56	85.06	87	132.88
328	chr4_000001F CDS g503	3	4.18E-32	chr_401_(-1)	89.41	92.98	85	122.86
329	chr4_000001F CDS g463	1	7.24E-26	chr_401_(-3)	77.11	78.31	83	101.29
330	chr4_000001F CDS g169	2	1.27E-18	chr_802_CDS_jgi.p_Aspni_NRRL3_1_11721	38.19	48.24	284	92.05
331	chr4_000001F CDS g916	1	4.41E-30	chr_402_(-2)	93.15	94.52	73	112.85
332	chr4_000001F CDS g1746	2	3.16E-39	chr_402_(-1)	89.74	93.59	249	148.67
333	chr4_000001F CDS g1913	2	1.78E-126	chr_701_(+1)	47.44	67.08	489	409.45
334	chr4_000001F CDS g1703	2	5.12E-03	chr_802_CDS_jgi.p_Aspni_NRRL3_1_11718	25.38	45.38	249	42.36
335	chr4_000001F CDS g1684	1	4.25E-30	chr_502_(-1)	37.79	51.74	168	117.47
336	chr4_000001F CDS g1660	2	1.19E-15	chr_202_(-3)	52.63	71.93	57	75.87
337	chr4_000001F CDS g1912	5	5.12E-81	chr_701_(+2)	41.67	60.55	552	289.27
338	chr4_000001F CDS g1699	2	6.46E-11	chr_102_CDS_jgi.p_Aspni_NRRL3_1_806	26.37	43.51	262	68.55
339	chr4_000001F CDS g1709	1	4.59E-03	chr_802_(+2)	38.18	60	53	38.89

340	chr4_000001F CDS g1698	2	2.30E-12	chr_802_(-3)	33.33	54.9	157	66.63
341	chr4_000001F CDS g1700	1	4.43E-11	chr_502_(-1)	37.33	57.33	75	62
342	chr4_000001F CDS g1784	2	4.61E-26	chr_402_(-3)	82.09	89.55	131	108.23
343	chr4_000001F CDS g1665	2	1.75E-09	chr_601_CDS_jgi.p_Aspni_NRRL3_1_8309	47.37	68.42	266	59.31
344	chr4_000001F CDS g1666	2	9.66E-08	chr_502_CDS_jgi.p_Aspni_NRRL3_1_7278	37.74	62.26	68	56.23
345	chr4_000001F CDS g1682	2	2.95E-43	chr_702_(+2)	29.93	48.63	389	168.7
346	chr4_000001F CDS g1674	2	1.06E-06	chr_501_CDS_NRRL3_06377-1	29.52	47.62	105	49.29
347	chr4_000001F CDS g1541	1	1.00E-47	chr_402_(+2)	85.25	86.07	122	165.24
348	chr4_000011F CDS g2447	1	2.80E-72	chr_401_(+3)	75.27	75.27	186	236.88
349	chr4_000011F CDS g2245	2	1.87E-06	chr_401_(-1)	26.67	54.44	170	49.68
350	chr4_000011F CDS g2250	1	5.73E-12	chr_502_(+2)	54	82	50	64.7
351	chr4_000011F CDS g2249	2	8.72E-26	chr_702_CDS_jgi.p_Aspni_NRRL3_1_9670	30.26	48.73	253	107.46
352	chr4_000011F CDS g2382	1	1.75E-26	chr_401_(+1)	66.35	67.31	89	102.83
353	chr4_000011F CDS g2264	3	4.62E-04	chr_502_(-2)	54.05	72.97	136	42.36
354	chr4_000011F CDS g2246	2	1.92E-14	chr_601_(-3)	26.76	54.93	208	75.48
355	chr4_000011F CDS g2256	2	3.85E-17	chr_702_(+3)	65.12	79.07	109	80.49
356	chr4_000011F CDS g2244	2	1.90E-07	chr_502_(-3)	41.51	61.6	124	57.77
357	chr4_000011F CDS g2221	2	7.22E-10	chr_502_CDS_jgi.p_Aspni_NRRL3_1_7130	28.87	46.48	139	60.85
358	chr4_000011F CDS g2248	2	5.78E-22	chr_402_CDS_jgi.p_Aspni_NRRL3_1_5804	29.11	42.73	218	92.82
359	chr4_000011F CDS g2247	1	3.07E-75	chr_502_(-1)	51.9	69.2	237	249.98
360	chr4_000011F CDS g2226	2	3.82E-14	chr_502_(+2)	68.09	76.6	53	68.17
361	chr4_000011F CDS g2241	2	2.60E-80	chr_502_(-1)	69.79	71.35	192	261.15
362	chr4_000011F CDS g2225	1	2.67E-24	chr_502_(-3)	42.5	65	120	98.98
363	chr5_000007F CDS g8508	2	2.05E-07	chr_502_(-3)	41.51	61.6	124	57.38
364	chr5_000007F CDS g8492	2	6.24E-46	chr_502_CDS_jgi.p_Aspni_NRRL3_1_6815	29.11	49.62	379	169.09
365	chr5_000007F CDS g8498	2	5.62E-23	chr_601_(-2)	50.46	63.3	130	97.44
366	chr5_000007F CDS g8535	2	2.97E-33	chr_601_(+1)	52.76	74.8	127	129.41
367	chr5_000007F CDS g8385	2	2.20E-29	chr_801_CDS_jgi.p_Aspni_NRRL3_1_10179	33.01	55.34	204	114.39
368	chr5_000007F CDS g8383	2	1.34E-14	chr_801_CDS_jgi.p_Aspni_NRRL3_1_10342	26.5	46.5	196	79.72
369	chr5_000007F CDS g8527	2	0	chr_202_(-3)	85.27	87.44	414	632.1
370	chr5_000007F CDS g8551	2	5.42E-09	chr_802_CDS_jgi.p_Aspni_NRRL3_1_11721	32.89	44.74	134	58.15
371	chr5_000007F CDS g8509	2	8.58E-12	chr_502_CDS_jgi.p_Aspni_NRRL3_1_7176	32.2	49.15	116	62
372	chr5_000007F CDS g8525	2	9.66E-17	chr_101_CDS_jgi.p_Aspni_NRRL3_1_152	29.33	46.67	201	87.04
373	chr5_000007F CDS g8528	3	1.25E-03	chr_102_(-2)	24.44	44.55	239	44.28
374	chr5_000007F CDS g8541	2	1.08E-09	chr_502_CDS_jgi.p_Aspni_NRRL3_1_7212	23.61	43.52	198	58.92
375	chr5_000007F CDS g8510	2	8.87E-19	chr_601_(-3)	73.33	83.33	191	84.73
376	chr5_000007F CDS g8542	2	7.25E-13	chr_601_(-3)	67.92	75.47	68	68.94
377	chr5_000007F CDS g8536	2	7.03E-45	chr_601_(-2)	61.74	75.65	243	163.31

378	chr5_000007F CDS g8484	3	6.42E-65	chr_601_(+3)	85.57	89.69	183	226.48
379	chr5_000007F CDS g8515	2	3.68E-21	chr_601_(+1)	77.78	87.04	62	90.89
380	chr5_000007F CDS g8497	2	2.62E-70	chr_601_(+3)	40.34	57.77	497	246.13
381	chr5_000007F CDS g8386	2	4.01E-18	chr_701_CDS_jgi.p_Aspni_NRRL3_1_8839	26.19	44.78	329	87.43
382	chr5_000007F CDS g8499	2	3.22E-53	chr_601_(-1)	47.87	62.5	328	192.2
383	chr5_000007F CDS g8526	1	4.41E-03	chr_601_CDS_jgi.p_Aspni_NRRL3_1_8719	25.18	47.48	139	40.43
384	chr5_000007F CDS g8539	2	5.67E-31	chr_601_(+1)	45.95	64.86	146	120.17
385	chr5_000007F CDS g8485	2	4.92E-07	chr_502_(-3)	41.51	61.6	124	57
386	chr5_000007F CDS g8546	2	5.22E-32	chr_601_(+1)	43.11	66.47	167	124.79
387	chr5_000007F CDS g8442	2	1.04E-54	chr_502_(-1)	70.45	75.68	132	186.04
388	chr5_000007F CDS g8544	3	1.17E-25	chr_601_(-3)	83.67	88.89	66	114
389	chr5_000007F CDS g8006	2	1.16E-147	chr_501_(+3)	93.88	93.88	245	461.84
390	chr5_000007F CDS g8487	2	1.31E-10	chr_601_CDS_jgi.p_Aspni_NRRL3_1_8652	37.25	52.94	112	59.69
391	chr5_000007F CDS g8518	2	1.27E-52	chr_601_(+1)	55.26	71.05	300	191.81
392	chr5_000008F CDS g7525	2	2.30E-05	chr_502_CDS_jgi.p_Aspni_NRRL3_1_7193	37.1	57.14	62	48.91
393	chr5_000008F CDS g7539	1	9.47E-21	chr_601_(+1)	51.11	63.33	90	87.81
394	chr5_000008F CDS g7617	2	4.32E-45	chr_601_(+1)	79.63	86.11	108	157.15
395	chr5_000008F CDS g7563	2	7.94E-07	chr_201_(+2)	51.35	59.46	79	49.29
396	chr5_000008F CDS g7704	2	2.01E-22	chr_601_(+3)	74.24	77.27	64	95.52
397	chr5_000008F CDS g7870	2	6.93E-24	chr_601_CDS_jgi.p_Aspni_NRRL3_1_7638	38.97	52.94	130	95.13
398	chr5_000008F CDS g7851	2	3.16E-14	chr_601_(-3)	66.04	73.58	68	73.17
399	chr5_000008F CDS g7764	1	2.06E-10	chr_601_(+1)	44.12	58.82	68	59.69
400	chr5_000008F CDS g7486	2	7.61E-07	chr_401_CDS_jgi.p_Aspni_NRRL3_1_5028	34	68	76	50.06
401	chr5_000008F CDS g7746	3	3.94E-130	chr_601_(+1)	78.72	89.36	309	426.79
402	chr5_000008F CDS g7619	1	1.38E-19	chr_601_(+1)	68.33	78.33	60	83.57
403	chr5_000008F CDS g7883	3	2.58E-17	chr_601_(-3)	64	73.08	75	82.42
404	chr5_000008F CDS g7648	3	1.55E-120	chr_701_(+1)	45.3	65.41	560	422.55
405	chr5_000008F CDS g7765	1	4.77E-20	chr_601_(+1)	49.48	65.98	97	85.11
406	chr5_000008F CDS g7535	2	1.00E-14	chr_702_CDS_jgi.p_Aspni_NRRL3_1_9400	48.98	75.51	63	71.25
407	chr5_000008F CDS g7658	2	4.18E-67	chr_601_(+3)	38.87	56.51	497	236.5
408	chr5_000008F CDS g7661	2	1.90E-07	chr_502_(-3)	41.51	61.6	124	57.77
409	chr5_000008F CDS g7789	2	1.61E-63	chr_601_(+3)	38.66	55.88	497	226.1
410	chr5_000008F CDS g7698	2	3.44E-30	chr_601_(+1)	42.17	65.06	166	119.4
411	chr5_000008F CDS g7506	2	5.71E-22	chr_401_CDS_jgi.p_Aspni_NRRL3_1_5028	41.75	64.08	126	92.82
412	chr5_000008F CDS g7495	3	5.86E-39	chr_702_CDS_jgi.p_Aspni_NRRL3_1_9400	38.98	56.28	244	149.06
413	chr5_000008F CDS g7474	2	4.87E-55	chr_801_(-2)	40.93	55.58	430	206.07
414	chr5_000008F CDS g7641	2	1.18E-04	chr_102_(-2)	25.64	48.72	156	47.75
415	chr5_000008F CDS g7659	2	5.34E-24	chr_601_(-2)	51.38	63.3	130	100.14

416	chr5_000008F CDS g7598	2	1.39E-16	chr_101_CDS_jgi.p_Aspni_NRRL3_1_152	28.85	46.22	201	86.66
417	chr5_000008F CDS g7565	2	8.72E-29	chr_202_(-1)	38.98	57.06	222	116.32
418	chr5_000008F CDS g7615	2	1.67E-118	chr_601_(+1)	64.06	75.62	320	383.26
419	chr5_000008F CDS g7869	2	3.09E-63	chr_601_(+3)	38.66	55.67	477	228.02
420	chr5_000008F CDS g7578	2	1.74E-22	chr_401_CDS_jgi.p_Aspni_NRRL3_1_5028	41.75	64.08	126	94.36
421	chr5_000008F CDS g7655	2	2.93E-31	chr_601_(+1)	42.77	65.66	166	122.48
422	chr5_000008F CDS g7794	2	2.06E-04	chr_301_(-1)	51.35	72.97	94	44.67
423	chr5_000008F CDS g7604	2	6.47E-11	chr_601_(-3)	69.23	79.49	53	63.93
424	chr5_000008F CDS g7649	2	3.60E-15	chr_601_(-3)	71.43	80	68	75.87
425	chr5_000008F CDS g7635	2	1.37E-52	chr_702_CDS_jgi.p_Aspni_NRRL3_1_9400	42.78	64.17	187	174.87
426	chr5_000008F CDS g7854	3	5.80E-16	chr_601_(-1)	88.89	94.44	68	78.18
427	chr5_000008F CDS g7599	1	4.45E-03	chr_601_CDS_jgi.p_Aspni_NRRL3_1_8719	25.18	47.48	139	40.43
428	chr5_000008F CDS g7637	1	2.20E-03	chr_502_CDS_jgi.p_Aspni_NRRL3_1_7193	25	43.28	256	43.13
429	chr5_000008F CDS g7595	5	5.87E-08	chr_102_(-2)	25	50	204	58.54
430	chr5_000008F CDS g7507	2	1.06E-42	chr_502_CDS_jgi.p_Aspni_NRRL3_1_7164	27.49	42.44	865	171.4
431	chr5_000008F CDS g7523	2	1.33E-17	chr_301_CDS_jgi.p_Aspni_NRRL3_1_2914	28.78	48.78	293	83.57
432	chr5_000008F CDS g7768	1	4.38E-13	chr_601_(-3)	23.79	43.17	206	72.02
433	chr5_000008F CDS g7611	1	7.64E-08	chr_601_(+2)	56.6	67.92	53	54.68
434	chr5_000008F CDS g7881	2	7.37E-27	chr_202_(-1)	36.16	54.8	222	110.92
435	chr5_000008F CDS g7527	1	2.42E-15	chr_802_(+2)	43.24	63.06	111	74.33
436	chr5_000008F CDS g7556	2	2.53E-07	chr_401_CDS_jgi.p_Aspni_NRRL3_1_5028	36	68	76	51.22
437	chr5_000008F CDS g7736	2	2.21E-47	chr_202_(-2)	72.41	79.31	116	170.63
438	chr5_000008F CDS g7510	2	7.14E-13	chr_601_(-1)	70.83	85.42	76	68.17
439	chr5_000008F CDS g7806	5	1.06E-05	chr_102_(-2)	25	49.44	200	51.22
440	chr5_000008F CDS g7577	2	3.27E-26	chr_601_(+2)	60.2	72.45	150	116.7
441	chr5_000008F CDS g7732	2	4.15E-28	chr_202_(-1)	28.7	41.45	447	117.86
442	chr5_000008F CDS g7734	2	1.47E-23	chr_601_(-2)	51.38	63.3	130	98.98
443	chr5_000008F CDS g7628	2	1.65E-18	chr_502_CDS_jgi.p_Aspni_NRRL3_1_7176	31.91	50.35	141	80.88
444	chr5_000008F CDS g7829	1	4.32E-05	chr_502_(-1)	48.84	62.79	43	46.21
445	chr5_000008F CDS g7800	2	1.09E-03	chr_201_(+2)	48.78	60.98	41	41.2
446	chr5_000008F CDS g7861	3	2.89E-46	chr_601_(+2)	79.07	86.05	171	167.93
447	chr5_000008F CDS g7380	1	2.73E-39	chr_601_(-3)	64.36	76.24	100	141.35
448	chr5_000008F CDS g7892	1	6.34E-116	chr_601_(+1)	61.1	72.05	347	374.01
449	chr5_000008F CDS g7887	3	2.73E-27	chr_601_(-3)	87.04	92.59	68	110.92
450	chr5_000008F CDS g7787	2	9.47E-59	chr_601_(-2)	53.09	68.72	243	202.99
451	chr5_000008F CDS g7667	2	6.43E-09	chr_302_CDS_jgi.p_Aspni_NRRL3_1_3854	46.88	56.25	64	54.3
452	chr5_000008F CDS g7733	2	3.84E-06	chr_601_(-1)	75.86	93.1	29	51.22
453	chr5_000008F CDS g7766	2	6.69E-25	chr_601_(+1)	41.57	63.41	166	104.38

454	chr5_000008F CDS g7740	2	2.56E-09	chr_601_(+3)	69.23	79.49	110	57.77
455	chr5_000008F CDS g7835	12	1.06E-09	chr_102_CDS_jgi.p_Aspni_NRRL3_1_765	38.55	62.2	118	58.92
456	chr5_000008F CDS g7644	2	3.44E-147	chr_601_(+1)	69.1	80.17	343	467.62
457	chr5_000008F CDS g7593	2	2.42E-32	chr_601_(+1)	43.71	65.87	167	125.56
458	chr5_000008F CDS g7845	2	3.16E-14	chr_601_(-3)	66.04	73.58	68	73.17
459	chr5_000008F CDS g7772	2	6.26E-09	chr_502_(-2)	38.98	61.02	59	53.14
460	chr5_000008F CDS g7815	2	1.11E-57	chr_102_CDS_jgi.p_Aspni_NRRL3_1_822	28.25	47.34	635	208.76
461	chr5_000008F CDS g7888	2	3.70E-07	chr_502_CDS_jgi.p_Aspni_NRRL3_1_7176	29.11	53.16	79	49.29
462	chr5_000008F CDS g7756	2	1.79E-19	chr_601_(-3)	88.68	94.34	53	93.2
463	chr5_000008F CDS g7880	2	1.98E-07	chr_502_(-3)	41.51	61.6	124	57.38
464	chr5_000008F CDS g7613	1	8.16E-22	chr_601_(+1)	50.31	56.44	163	101.68
465	chr5_000008F CDS g7668	2	1.33E-15	chr_202_CDS_jgi.p_Aspni_NRRL3_1_2020	32.71	47.66	203	80.11
466	chr5_000008F CDS g7631	2	8.48E-09	chr_502_CDS_jgi.p_Aspni_NRRL3_1_7212	24.59	42.62	166	56.61
467	chr5_000008F CDS g7877	2	4.02E-23	chr_601_(+3)	75.76	77.27	64	95.9
468	chr5_000008F CDS g7761	2	1.22E-11	chr_502_(+1)	51.28	66.67	96	70.09
469	chr5_000008F CDS g7747	2	3.47E-129	chr_601_(+1)	69.05	76.79	385	417.54
470	chr5_000008F CDS g7786	2	2.25E-30	chr_601_(+1)	42.6	65.68	167	120.17
471	chr5_000008F CDS g7490	1	2.26E-04	chr_502_(-1)	35.62	54.79	73	43.9
472	chr5_000008F CDS g7627	3	2.32E-28	chr_601_(-3)	83.67	88.89	66	114
473	chr5_000008F CDS g7642	2	1.09E-45	chr_102_CDS_jgi.p_Aspni_NRRL3_1_822	28.94	45.42	525	176.41
474	chr5_000008F CDS g7714	2	3.56E-13	chr_601_(-3)	64.15	75.47	68	70.09
475	chr5_000008F CDS g7711	3	4.33E-27	chr_601_(-3)	83.67	87.04	66	110.15
476	chr5_000008F CDS g7654	2	7.06E-08	chr_601_(-3)	52.83	67.92	65	56.61
477	chr5_000008F CDS g7656	2	4.45E-46	chr_601_(-2)	54.59	67.63	207	164.85
478	chr5_000008F CDS g7741	1	6.83E-32	chr_601_(+1)	45.98	54.6	165	122.48
479	chr5_000008F CDS g7676	2	1.38E-26	chr_601_(+1)	87.04	90.74	60	106.3
480	chr5_000008F CDS g7797	1	8.51E-11	chr_601_(+1)	45.33	61.33	74	63.16
481	chr5_000008F CDS g7726	2	9.57E-17	chr_101_CDS_jgi.p_Aspni_NRRL3_1_152	29.33	46.67	201	87.04
482	chr5_000008F CDS g7464	1	4.52E-68	chr_202_(+2)	84.21	84.21	152	224.56
483	chr5_000008F CDS g7866	2	2.12E-36	chr_601_(+1)	49.04	70.06	157	136.73
484	chr5_000008F CDS g7727	1	2.13E-03	chr_601_CDS_jgi.p_Aspni_NRRL3_1_8719	25.18	48.2	139	41.59
485	chr5_000008F CDS g7528	2	4.26E-38	chr_502_(+3)	43.48	62.61	229	143.28
486	chr5_000008F CDS g7660	2	2.07E-143	chr_601_(-1)	55.47	68.79	503	458.37
487	chr5_000008F CDS g7630	2	1.48E-11	chr_601_(-3)	66.04	73.58	68	65.47
488	chr5_000008F CDS g7716	1	5.21E-26	chr_601_(+1)	49.66	62.42	148	107.46
489	chr5_000008F CDS g7853	2	4.81E-42	chr_601_(+3)	73.21	82.14	111	161
490	chr5_000008F CDS g7480	2	3.47E-13	chr_601_(-3)	27.4	53.42	185	72.02
491	chr5_000008F CDS g7699	2	2.28E-60	chr_601_(-2)	54.32	70.78	243	207.61

492	chr5_000008F CDS g7579	2	1.34E-45	chr_502_CDS_jgi.p_Aspni_NRRL3_1_7164	28.04	42.23	865	180.26
493	chr5_000008F CDS g7792	2	5.77E-17	chr_601_(-2)	31.3	58.02	206	87.81
494	chr5_000008F CDS g7785	2	1.09E-08	chr_601_(-3)	54.72	69.81	65	59.31
495	chr5_000008F CDS g7809	2	9.41E-17	chr_101_CDS_jgi.p_Aspni_NRRL3_1_152	29.33	46.67	201	87.04
496	chr5_000008F CDS g7791	3	2.16E-26	chr_402_(-2)	84.85	93.94	119	112.85
497	chr5_000008F CDS g7616	1	4.51E-32	chr_601_(+1)	46.2	62.66	158	123.25
498	chr5_000008F CDS g7731	2	9.10E-67	chr_102_CDS_jgi.p_Aspni_NRRL3_1_822	29.24	47.88	624	238.04
499	chr5_000008F CDS g7749	2	4.67E-16	chr_502_(+1)	50.79	71.43	74	81.26
500	chr5_000008F CDS g7811	2	2.70E-15	chr_601_CDS_jgi.p_Aspni_NRRL3_1_8719	43.84	65.75	73	68.17
501	chr5_000008F CDS g7867	2	9.73E-52	chr_601_(-2)	61.74	76.52	243	182.96
502	chr5_000008F CDS g7750	2	5.04E-05	chr_502_CDS_jgi.p_Aspni_NRRL3_1_6646	30.56	44.44	109	45.82
503	chr5_000008F CDS g7646	1	5.17E-44	chr_601_(+3)	72.97	74.77	111	161.38
504	chr5_000008F CDS g7570	1	9.53E-10	chr_402_CDS_jgi.p_Aspni_NRRL3_1_6136	35.48	52.69	92	61.62
505	chr5_000008F CDS g7686	1	1.57E-29	chr_601_(+1)	45.2	55.37	165	118.63
506	chr5_000008F CDS g7609	2	7.04E-05	chr_502_CDS_jgi.p_Aspni_NRRL3_1_7196	34.67	60	71	45.05
507	chr5_000008F CDS g7505	2	2.94E-25	chr_601_(+2)	55.05	67.89	150	113.62
508	chr5_000008F CDS g7745	2	8.28E-13	chr_601_(-3)	64.15	73.58	65	72.79
509	chr5_000008F CDS g7712	2	1.51E-15	chr_502_CDS_jgi.p_Aspni_NRRL3_1_7176	30.5	48.23	141	73.17
510	chr5_000008F CDS g7790	2	3.93E-23	chr_601_(-2)	49.54	62.39	130	97.83
511	chr5_000008F CDS g7559	1	2.12E-04	chr_502_(-1)	35.62	54.79	73	43.9
512	chr5_000008F CDS g7603	2	2.40E-49	chr_102_CDS_jgi.p_Aspni_NRRL3_1_822	27.49	44.85	518	182.57
513	chr6_000005F CDS g2728	2	1.74E-22	chr_401_CDS_jgi.p_Aspni_NRRL3_1_5028	41.75	64.08	126	94.36
514	chr6_000005F CDS g2718	2	6.28E-33	chr_702_CDS_jgi.p_Aspni_NRRL3_1_9539	28.57	45.45	437	134.03
515	chr6_000005F CDS g2700	3	1.25E-03	chr_102_(-2)	24.44	44.55	239	44.28
516	chr6_000005F CDS g2698	3	1.05E-106	chr_601_(-2)	60.35	74.04	281	342.81
517	chr6_000005F CDS g2747	1	2.12E-04	chr_502_(-1)	35.62	54.79	73	43.9
518	chr6_000005F CDS g2750	2	2.53E-07	chr_401_CDS_jgi.p_Aspni_NRRL3_1_5028	36	68	76	51.22
519	chr6_000005F CDS g2740	2	2.41E-06	chr_302_CDS_jgi.p_Aspni_NRRL3_1_3638	32	50.67	77	52.76
520	chr6_000005F CDS g2729	2	3.27E-26	chr_601_(+2)	60.2	72.45	150	116.7
521	chr6_000005F CDS g2720	2	3.00E-24	chr_601_CDS_jgi.p_Aspni_NRRL3_1_7615	30.86	45.23	232	108.23
522	chr6_000005F CDS g2783	2	2.96E-30	chr_701_CDS_jgi.p_Aspni_NRRL3_1_9062	32.33	51.13	250	125.95
523	chr6_000005F CDS g2701	2	0	chr_202_(-3)	85.27	87.44	414	632.1
524	chr6_000005F CDS g2769	2	1.05E-74	chr_601_CDS_jgi.p_Aspni_NRRL3_1_8386	34.41	50.58	398	241.89
525	chr6_000005F CDS g2743	2	2.00E-25	chr_202_(-1)	36.67	55	222	112.46
526	chr6_000005F CDS g2713	2	1.98E-07	chr_502_(-3)	41.51	61.6	124	57.38
527	chr6_000005F CDS g2762	3	4.42E-133	chr_601_(-1)	91.95	94.25	244	422.17
528	chr6_000005F CDS g2702	1	3.20E-03	chr_601_CDS_jgi.p_Aspni_NRRL3_1_8719	25.18	47.48	139	40.82
529	chr6_000005F CDS g2727	2	1.34E-45	chr_502_CDS_jgi.p_Aspni_NRRL3_1_7164	28.04	42.23	865	180.26

530	chr6_000005F CDS g3161	2	1.73E-14	chr_301_CDS_jgi.p_Aspni_NRRL3_1_3242	30	51.67	120	75.87
531	chr6_000005F CDS g2806	1	5.91E-03	chr_202_CDS_jgi.p_Aspni_NRRL3_1_2840	27.4	38.81	202	40.82
532	chr6_000005F CDS g2714	3	6.42E-65	chr_601_(+3)	85.57	89.69	183	226.48
533	chr6_000005F CDS g2809	2	3.91E-11	chr_702_CDS_jgi.p_Aspni_NRRL3_1_9427	23.13	45.93	350	69.32
534	chr6_000005F CDS g2703	2	9.66E-17	chr_101_CDS_jgi.p_Aspni_NRRL3_1_152	29.33	46.67	201	87.04
535	chr6_000005F CDS g2721	2	3.45E-33	chr_502_CDS_jgi.p_Aspni_NRRL3_1_7153	40.61	56.85	195	139.04
536	chr6_000005F CDS g2712	2	1.27E-52	chr_601_(+1)	55.26	71.05	300	191.81
537	chr6_000005F CDS g2741	2	1.98E-07	chr_502_(-3)	41.51	61.6	124	57.38
538	chr6_000005F CDS g2722	2	5.16E-14	chr_502_(-3)	30.06	45.66	193	75.1
539	chr6_000005F CDS g3308	2	9.10E-11	chr_802_CDS_NRRL3_10811-1	27.52	48.32	149	62.77
540	chr6_000005F CDS g3825	2	4.47E-109	chr_302_(-1)	67.69	75.38	401	358.22
541	chr6_000005F CDS g3314	2	4.84E-71	chr_601_(+1)	86.76	92.65	138	236.5
542	chr6_000005F CDS g3352	1	2.39E-74	chr_601_(-2)	87.8	87.8	164	243.82
543	chr6_000005F CDS g3808	2	1.90E-29	chr_601_(-2)	86.21	86.21	114	111.69
544	chr6_000005F CDS g3840	4	4.15E-103	chr_601_(-1)	91.54	93.53	201	331.26
545	chr6_000005F CDS g3307	1	9.24E-06	chr_301_(+1)	21.94	41.94	152	47.37
546	chr7_000002F CDS g8685	2	1.46E-11	chr_701_(-1)	34.81	48.1	155	63.93
547	chr7_000002F CDS g9041	2	5.09E-11	chr_702_(-3)	42.03	66.67	170	66.63
548	chr7_000002F CDS g9167	2	3.07E-04	chr_502_CDS_jgi.p_Aspni_NRRL3_1_7196	28.89	45.93	134	46.21
549	chr7_000002F CDS g9169	5	2.90E-17	chr_601_(-3)	34.38	51.33	187	89.35
550	chr7_000002F CDS g9157	2	1.26E-44	chr_202_(-3)	37.38	56.63	308	172.94
551	chr7_000002F CDS g8736	2	5.27E-72	chr_701_(+3)	85.92	86.62	142	235.34
552	chr7_000002F CDS g8625	1	3.65E-60	chr_701_(-3)	82.54	82.54	126	200.29
553	chr7_000002F CDS g9168	2	1.45E-06	chr_402_CDS_NRRL3_05106	23.94	42.72	200	53.14
554	chr7_000002F CDS g9195	2	5.90E-44	chr_702_CDS_jgi.p_Aspni_NRRL3_1_9382	48.61	59.72	256	151.75
555	chr7_000002F CDS g8563	1	3.86E-35	chr_502_(-1)	50.85	71.19	113	132.88
556	chr7_000002F CDS g9158	2	9.90E-07	chr_502_CDS_jgi.p_Aspni_NRRL3_1_7153	27.89	44.21	175	54.3
557	chr7_000002F CDS g8630	1	5.05E-66	chr_701_(+3)	92.31	93.16	117	217.62
558	chr7_000002F CDS g9304	2	3.64E-12	chr_801_CDS_jgi.p_Aspni_NRRL3_1_10445	23.81	40.14	288	71.25
559	chr7_000002F CDS g9548	1	9.53E-46	chr_702_(-3)	94.19	94.19	86	157.92
560	chr7_000002F CDS g9404	3	6.34E-47	chr_702_(-1)	80.77	91.35	118	177.95
561	chr8_000003F CDS g11414	1	3.80E-112	chr_802_(+2)	88.44	88.44	199	353.6
562	chr8_000003F CDS g11046	2	6.11E-30	chr_802_(-2)	85.71	91.43	60	114.39
563	chr8_000003F CDS g11588	3	1.65E-33	chr_502_(-2)	55.56	80	365	135.19
564	chr8_000003F CDS g12040	1	2.32E-121	chr_801_(+1)	90.41	90.87	219	380.56
565	chr8_000009F CDS g6018	3	5.05E-22	chr_702_(+3)	70.91	89.09	55	92.82
566	chr8_000009F CDS g6041	2	1.16E-46	chr_601_(+1)	31.93	50.25	381	174.1
567	scaffold1_000010F CDS g2204	4	9.20E-31	chr_402_(-2)	80.25	87.93	81	125.56

568	scaffold1_000010F CDS g1982	2	2.76E-07	chr_801_(+3)	27.62	43.09	179	53.91
569	scaffold1_000010F CDS g2152	2	2.56E-71	chr_601_(+2)	73.78	73.78	164	234.19
570	scaffold2_000012F CDS g2602	1	1.17E-15	chr_601_(+1)	52.27	60.61	132	85.11
571	scaffold2_000012F CDS g2503	2	9.04E-69	chr_601_(+1)	43.9	60.98	504	241.12
572	scaffold2_000012F CDS g2509	2	8.34E-18	chr_502_CDS_jgi.p_Aspni_NRRL3_1_7176	31.21	49.65	141	79.34
573	scaffold2_000012F CDS g2539	2	7.65E-27	chr_202_(-1)	28.2	41.28	447	114.39
574	scaffold2_000012F CDS g2621	2	9.09E-54	chr_601_(+3)	49.58	68.91	244	199.13
575	scaffold2_000012F CDS g2477	3	2.58E-17	chr_601_(-3)	64	73.08	75	82.42
576	scaffold2_000012F CDS g2547	2	2.07E-05	chr_502_CDS_NRRL3_07196-2	28.89	44.44	171	48.14
577	scaffold2_000012F CDS g2576	2	1.85E-71	chr_601_(+3)	39.08	56.3	497	249.21
578	scaffold2_000012F CDS g2501	3	2.28E-176	chr_601_(+1)	78.91	83.59	506	553.13
579	scaffold2_000012F CDS g2567	2	3.94E-23	chr_601_(+3)	75.76	78.79	64	97.83
580	scaffold2_000012F CDS g2491	3	2.89E-46	chr_601_(+2)	79.07	86.05	171	167.93
581	scaffold2_000012F CDS g2563	2	3.01E-12	chr_601_(-3)	64.15	73.58	68	67.4
582	scaffold2_000012F CDS g2485	2	6.11E-25	chr_601_(-2)	49.57	61.54	125	103.22
583	scaffold2_000012F CDS g2550	3	9.27E-18	chr_601_(-1)	88.89	94.44	68	83.19
584	scaffold2_000012F CDS g2585	2	1.25E-05	chr_202_CDS_jgi.p_Aspni_NRRL3_1_1847	37.1	54.84	98	43.13
585	scaffold2_000012F CDS g2469	1	2.20E-105	chr_601_(+1)	57.35	67.44	347	342.81
586	scaffold2_000012F CDS g2605	1	2.70E-05	chr_802_(+2)	64.1	71.79	39	48.14
587	scaffold2_000012F CDS g2502	3	7.36E-112	chr_601_(+1)	80.85	89.36	309	367.08
588	scaffold2_000012F CDS g2613	3	2.53E-28	chr_601_(-3)	83.67	88.89	66	113.62
589	scaffold2_000012F CDS g2606	12	7.76E-10	chr_102_CDS_jgi.p_Aspni_NRRL3_1_765	38.55	62.2	118	59.31
590	scaffold2_000012F CDS g2554	1	4.91E-32	chr_601_(+1)	41.75	55.34	199	125.95
591	scaffold2_000012F CDS g2559	2	3.72E-09	chr_302_CDS_jgi.p_Aspni_NRRL3_1_3854	55.1	67.35	49	53.14
592	scaffold2_000012F CDS g2531	2	3.48E-06	chr_301_CDS_NRRL3_03536-1	66.67	76.92	181	51.22
593	scaffold2_000012F CDS g2588	2	1.81E-13	chr_502_(+3)	25.1	44.03	242	73.17
594	scaffold2_000012F CDS g2472	2	3.70E-07	chr_502_CDS_jgi.p_Aspni_NRRL3_1_7176	29.11	53.16	79	49.29
595	scaffold2_000012F CDS g2555	1	6.80E-20	chr_601_(+1)	48.39	61.29	93	86.27
596	scaffold2_000012F CDS g2572	2	7.99E-29	chr_601_(+1)	41.32	64.07	167	115.55
597	scaffold2_000012F CDS g2504	3	1.85E-17	chr_601_(-3)	64	73.08	75	82.8
598	scaffold2_000012F CDS g2548	2	2.10E-68	chr_601_(-3)	85.71	88.72	133	226.87
599	scaffold2_000012F CDS g2499	2	8.95E-22	chr_601_(+2)	85.19	90.74	71	92.82
600	scaffold2_000012F CDS g2527	2	1.79E-42	chr_601_(+1)	50.6	70.83	168	154.84
601	scaffold2_000012F CDS g2479	2	7.37E-27	chr_202_(-1)	36.16	54.8	222	110.92
602	scaffold2_000012F CDS g2546	4	1.11E-07	chr_102_(-2)	24.49	48.47	315	57.38
603	scaffold2_000012F CDS g2492	2	8.37E-20	chr_601_(-3)	90.57	94.34	75	93.97
604	scaffold2_000012F CDS g2614	2	1.28E-17	chr_502_CDS_jgi.p_Aspni_NRRL3_1_7176	30.32	47.1	155	78.57
605	scaffold2_000012F CDS g2627	2	1.60E-32	chr_601_(+1)	43.11	67.07	167	126.33

606	scaffold2_000012F CDS g2626	2	2.03E-69	chr_601_(+1)	45.81	61.94	310	243.05
607	scaffold2_000012F CDS g2628	2	9.45E-46	chr_601_(-2)	54.59	67.63	207	164.08
608	scaffold2_000012F CDS g2533	2	5.01E-16	chr_601_(+1)	76.67	83.33	59	75.87
609	scaffold2_000012F CDS g2578	3	7.55E-21	chr_502_(+2)	26.74	43.31	320	97.44
610	scaffold2_000012F CDS g2544	1	4.01E-03	chr_601_CDS_jgi.p_Aspni_NRRL3_1_8719	25.18	47.48	139	40.43
611	scaffold2_000012F CDS g2483	2	1.35E-30	chr_702_CDS_jgi.p_Aspni_NRRL3_1_9539	29.76	46.34	414	127.1
612	scaffold2_000012F CDS g2540	2	3.98E-68	chr_102_CDS_jgi.p_Aspni_NRRL3_1_822	28.24	46.18	640	237.27
613	scaffold2_000012F CDS g2573	2	1.39E-59	chr_601_(-2)	53.91	69.96	243	205.3
614	scaffold2_000012F CDS g2486	2	1.09E-72	chr_601_(+3)	39.08	57.14	477	255.76
615	scaffold2_000012F CDS g2489	2	4.96E-38	chr_601_(+1)	48.52	69.88	169	142.12
616	scaffold2_000012F CDS g2537	1	2.86E-33	chr_601_(+1)	47.16	56.82	165	126.72
617	scaffold2_000012F CDS g2480	2	1.98E-07	chr_502_(-3)	41.51	61.6	124	57.38
618	scaffold2_000012F CDS g2592	2	5.69E-97	chr_601_(+1)	54.55	72.73	330	319.7
619	scaffold2_000012F CDS g2512	1	3.16E-115	chr_601_(+1)	46.11	57.59	511	374.4
620	scaffold2_000012F CDS g2508	3	2.73E-27	chr_601_(-3)	87.04	92.59	68	110.92
621	scaffold2_000012F CDS g2622	3	7.25E-13	chr_601_(-3)	63.46	73.08	68	68.94
622	scaffold2_000012F CDS g2594	2	8.14E-23	chr_601_(+1)	79.66	87.04	59	95.9
623	scaffold2_000012F CDS g2591	2	2.37E-08	chr_202_CDS_jgi.p_Aspni_NRRL3_1_2264	35.71	54.76	124	53.53
624	scaffold2_000012F CDS g2630	2	2.00E-64	chr_601_(+3)	38.66	56.72	476	231.49
625	scaffold2_000012F CDS g2473	3	2.73E-27	chr_601_(-3)	87.04	92.59	68	110.92
626	scaffold2_000012F CDS g2616	3	3.60E-15	chr_601_(-3)	64.71	73.08	68	75.87
627	scaffold2_000012F CDS g2601	2	1.05E-08	chr_601_(-3)	54.72	69.81	65	59.31
628	scaffold2_000012F CDS g2631	2	1.01E-23	chr_601_(-2)	51.38	62.39	130	99.37
629	scaffold2_000012F CDS g2514	2	4.05E-22	chr_601_(+2)	87.04	90.74	71	93.59
630	scaffold2_000012F CDS g2617	2	7.85E-71	chr_601_(+1)	62.96	74.07	469	245.36
631	scaffold2_000012F CDS g2488	2	9.73E-52	chr_601_(-2)	61.74	76.52	243	182.96
632	scaffold3_000013F CDS g110	2	8.17E-05	chr_202_CDS_jgi.p_Aspni_NRRL3_1_1847	33.33	45.71	109	40.82
633	scaffold3_000013F CDS g80	1	1.50E-07	chr_802_(+3)	71.88	90.62	32	52.76
634	scaffold3_000013F CDS g60	2	3.80E-18	chr_601_(-3)	64.2	70.83	81	88.58
635	scaffold3_000013F CDS g24	2	2.02E-07	chr_502_(-3)	41.51	61.6	124	57.38
636	scaffold3_000013F CDS g101	2	6.54E-10	chr_601_(-3)	58.49	71.7	65	62.77
637	scaffold3_000013F CDS g86	2	1.79E-19	chr_601_(-3)	88.68	94.34	53	93.2
638	scaffold3_000013F CDS g2	2	7.37E-27	chr_202_(-1)	36.16	54.8	222	110.92
639	scaffold3_000013F CDS g115	1	2.45E-14	chr_302_(-2)	55.84	64.94	77	79.72
640	scaffold3_000013F CDS g18	9	2.36E-10	chr_102_(+2)	39.53	62.2	158	60.85
641	scaffold3_000013F CDS g7	3	9.02E-29	chr_601_(-1)	82.69	86.54	159	122.86
642	scaffold3_000013F CDS g128	3	1.17E-25	chr_601_(-3)	83.67	88.89	66	114
643	scaffold3_000013F CDS g105	2	3.14E-06	chr_102_(+1)	39.73	52.05	87	46.98

644	scaffold3_000013F CDS g126	2	5.22E-32	chr_601_(+1)	43.11	66.47	167	124.79
645	scaffold3_000013F CDS g124	2	5.36E-04	chr_102_(-1)	24.73	50.54	78	41.2
646	scaffold3_000013F CDS g30	2	1.66E-74	chr_601_(+3)	39.29	57.35	477	261.15
647	scaffold3_000013F CDS g29	2	6.11E-25	chr_601_(-2)	49.57	61.54	125	103.22
648	scaffold3_000013F CDS g130	2	7.25E-13	chr_601_(-3)	67.92	75.47	68	68.94
649	scaffold3_000013F CDS g107	2	9.61E-19	chr_202_CDS_jgi.p_Aspni_NRRL3_1_1678	28.17	47.96	206	89.35
650	scaffold3_000013F CDS g79	2	1.03E-13	chr_601_(-1)	74.51	82.35	68	71.63
651	scaffold3_000013F CDS g11	1	6.34E-116	chr_601_(+1)	61.1	72.05	347	374.01
652	scaffold3_000013F CDS g1	2	2.58E-07	chr_502_(-3)	41.51	61.6	124	56.23
653	scaffold3_000013F CDS g56	1	4.97E-156	chr_601_(+1)	51.87	63.86	518	493.43
654	scaffold3_000013F CDS g120	2	5.42E-09	chr_802_CDS_jgi.p_Aspni_NRRL3_1_11721	32.89	44.74	134	58.15
655	scaffold3_000013F CDS g4	3	2.58E-17	chr_601_(-3)	64	73.08	75	82.42
656	scaffold3_000013F CDS g32	2	1.52E-36	chr_601_(-2)	63.64	75.32	202	137.5
657	scaffold3_000013F CDS g59	1	2.53E-06	chr_701_(+1)	19.12	42.38	385	55.07
658	scaffold3_000013F CDS g61	12	7.76E-10	chr_102_CDS_jgi.p_Aspni_NRRL3_1_765	38.55	62.2	118	59.31
659	scaffold3_000013F CDS g65	2	1.07E-19	chr_502_CDS_jgi.p_Aspni_NRRL3_1_7152	34.27	54.55	265	94.36
660	scaffold3_000013F CDS g27	2	1.35E-30	chr_702_CDS_jgi.p_Aspni_NRRL3_1_9539	29.76	46.34	414	127.1
661	scaffold3_000013F CDS g40	2	4.96E-52	chr_202_(+1)	45.64	69.23	195	192.59
662	scaffold3_000013F CDS g69	2	7.87E-22	chr_601_(+1)	77.97	83.05	64	93.2
663	scaffold3_000013F CDS g70	1	7.66E-10	chr_601_(+1)	34.26	50	101	57
664	scaffold3_000013F CDS g81	2	1.05E-04	chr_502_CDS_jgi.p_Aspni_NRRL3_1_6646	30	41.54	126	44.28
665	scaffold3_000013F CDS g19	2	3.62E-17	chr_601_(-3)	80.77	86.54	52	83.19
666	scaffold3_000013F CDS g33	2	5.36E-38	chr_601_(+1)	48.52	69.88	169	142.12
667	scaffold3_000013F CDS g46	2	8.37E-20	chr_601_(-3)	90.57	94.34	75	93.97
668	scaffold3_000013F CDS g53	2	1.18E-28	chr_601_(+2)	69.51	79.27	82	113.62
669	scaffold3_000013F CDS g76	2	3.22E-71	chr_601_(+1)	55.26	71.05	511	248.05
670	scaffold3_000013F CDS g82	2	6.55E-12	chr_502_(+1)	51.28	66.67	69	68.55
671	scaffold3_000013F CDS g57	3	1.06E-16	chr_601_(-1)	88.89	94.44	68	80.11
672	scaffold3_000013F CDS g92	2	1.40E-26	chr_601_(+1)	87.04	90.74	60	106.3
673	scaffold4_000014R CDS g2693	3	1.17E-25	chr_601_(-3)	83.67	88.89	66	114
674	scaffold4_000014R CDS g2661	2	3.09E-63	chr_601_(+3)	38.66	55.67	477	228.02
675	scaffold4_000014R CDS g2644	2	3.70E-07	chr_502_CDS_jgi.p_Aspni_NRRL3_1_7176	29.11	53.16	79	49.29
676	scaffold4_000014R CDS g2685	2	5.42E-09	chr_802_CDS_jgi.p_Aspni_NRRL3_1_11721	32.89	44.74	134	58.15
677	scaffold4_000014R CDS g2695	2	7.25E-13	chr_601_(-3)	67.92	75.47	68	68.94
678	scaffold4_000014R CDS g2638	2	7.37E-27	chr_202_(-1)	36.16	54.8	222	110.92
679	scaffold4_000014R CDS g2648	1	6.34E-116	chr_601_(+1)	61.1	72.05	347	374.01
680	scaffold4_000014R CDS g2640	3	2.58E-17	chr_601_(-3)	64	73.08	75	82.42
681	scaffold4_000014R CDS g2691	2	5.22E-32	chr_601_(+1)	43.11	66.47	167	124.79

682	scaffold4_000014R CDS g2637	2	2.58E-07	chr_502_(-3)	41.51	61.6	124	56.23
683	scaffold4_000014R CDS g2643	3	1.03E-21	chr_601_(-1)	87.04	92.59	54	93.97
684	scaffold4_000014R CDS g2677	9	2.36E-10	chr_102_(+2)	39.53	62.2	158	60.85
685	scaffold4_000014R CDS g2662	2	6.93E-24	chr_601_CDS_jgi.p_Aspni_NRRL3_1_7638	38.97	52.94	130	95.13
686	scaffold4_000014R CDS g2659	2	9.73E-52	chr_601_(-2)	61.74	76.52	243	182.96
687	scaffold4_000014R CDS g2689	2	5.36E-04	chr_102_(-1)	24.73	50.54	78	41.2
688	scaffold4_000014R CDS g2671	2	2.02E-07	chr_502_(-3)	41.51	61.6	124	57.38
689	scaffold4_000014R CDS g2658	2	4.96E-38	chr_601_(+1)	48.52	69.88	169	142.12
690	scaffold4_000014R CDS g2681	2	4.71E-06	chr_601_CDS_jgi.p_Aspni_NRRL3_1_7638	45.1	54.9	51	48.14
691	scaffold4_000014R CDS g2696	2	1.08E-09	chr_502_CDS_jgi.p_Aspni_NRRL3_1_7212	23.61	43.52	198	58.92
692	scaffold4_000014R CDS g2668	2	4.02E-23	chr_601_(+3)	75.76	77.27	64	95.9
693	scaffold4_000014R CDS g2656	3	2.89E-46	chr_601_(+2)	79.07	86.05	171	167.93
694	scaffold4_000014R CDS g2676	2	3.62E-17	chr_601_(-3)	80.77	86.54	52	83.19

Table S7. Unique protein sequences in the proteome of NRRL3 compared to the entire proteome of CBS 554.65 by a blastp analysis.

No	Query	Number of HSPs	Lowest E-value	Accession (E-value)	Greatest identity %	Greatest positive %	Greatest HSP length	Greatest bit score
1	jgi|Aspni_NRRL3_1|45|	0	no hit	not available	-	-	-	-
2	jgi|Aspni_NRRL3_1|1210|	0	no hit	not available	-	-	-	-
3	jgi|Aspni_NRRL3_1|1211|	0	no hit	not available	-	-	-	-
4	jgi|Aspni_NRRL3_1|1215|	0	no hit	not available	-	-	-	-
5	jgi|Aspni_NRRL3_1|1697|	0	no hit	not available	-	-	-	-
6	jgi|Aspni_NRRL3_1|1699|	0	no hit	not available	-	-	-	-
7	jgi|Aspni_NRRL3_1|1712|	0	no hit	not available	-	-	-	-
8	jgi|Aspni_NRRL3_1|3295|	0	no hit	not available	-	-	-	-
9	jgi|Aspni_NRRL3_1|3296|	0	no hit	not available	-	-	-	-
10	jgi|Aspni_NRRL3_1|4218|	0	no hit	not available	-	-	-	-
11	jgi|Aspni_NRRL3_1|4220|	0	no hit	not available	-	-	-	-
12	jgi|Aspni_NRRL3_1|4321|	0	no hit	not available	-	-	-	-
13	jgi|Aspni_NRRL3_1|4426|	0	no hit	not available	-	-	-	-
14	jgi|Aspni_NRRL3_1|5382|	0	no hit	not available	-	-	-	-
15	jgi|Aspni_NRRL3_1|5385|	0	no hit	not available	-	-	-	-
16	jgi|Aspni_NRRL3_1|5403|	0	no hit	not available	-	-	-	-
17	jgi|Aspni_NRRL3_1|5404|	0	no hit	not available	-	-	-	-
18	jgi|Aspni_NRRL3_1|5839|	0	no hit	not available	-	-	-	-
19	jgi|Aspni_NRRL3_1|6298|	0	no hit	not available	-	-	-	-
20	jgi|Aspni_NRRL3_1|7150|	0	no hit	not available	-	-	-	-

21	jgi|Aspni_NRRL3_1|7157|	0	no hit	not available	-	-	-	-
22	jgi|Aspni_NRRL3_1|7162|	0	no hit	not available	-	-	-	-
23	jgi|Aspni_NRRL3_1|7191|	0	no hit	not available	-	-	-	-
24	jgi|Aspni_NRRL3_1|7192|	0	no hit	not available	-	-	-	-
25	jgi|Aspni_NRRL3_1|7198|	0	no hit	not available	-	-	-	-
26	jgi|Aspni_NRRL3_1|7200|	0	no hit	not available	-	-	-	-
27	jgi|Aspni_NRRL3_1|7420|	0	no hit	not available	-	-	-	-
28	jgi|Aspni_NRRL3_1|7872|	0	no hit	not available	-	-	-	-
29	jgi|Aspni_NRRL3_1|8794|	0	no hit	not available	-	-	-	-
30	jgi|Aspni_NRRL3_1|9301|	0	no hit	not available	-	-	-	-
31	jgi|Aspni_NRRL3_1|9305|	0	no hit	not available	-	-	-	-
32	jgi|Aspni_NRRL3_1|9454|	0	no hit	not available	-	-	-	-
33	jgi|Aspni_NRRL3_1|9470|	0	no hit	not available	-	-	-	-
34	jgi|Aspni_NRRL3_1|9472|	0	no hit	not available	-	-	-	-
35	jgi|Aspni_NRRL3_1|9563|	0	no hit	not available	-	-	-	-
36	jgi|Aspni_NRRL3_1|9652|	0	no hit	not available	-	-	-	-
37	jgi|Aspni_NRRL3_1|9820|	0	no hit	not available	-	-	-	-
38	jgi|Aspni_NRRL3_1|17|	2	6.60E-64	chr1_000006F_(-3)	82.93	82.93	123	211.07
39	jgi|Aspni_NRRL3_1|50|	1	2.68E-06	chr1_000006F_(+3)	33.82	41.91	125	49.68
40	jgi|Aspni_NRRL3_1|77|	2	8.38E-119	chr1_000006F_(+1)	88.89	88.89	189	371.32
41	jgi|Aspni_NRRL3_1|114|	1	3.32E-04	chr2_000000F_(-3)	25.62	40.94	316	45.05
42	jgi|Aspni_NRRL3_1|165|	1	2.81E-10	chr3_000004F_(-3)	30.17	41.9	175	61.62
43	jgi|Aspni_NRRL3_1|215|	2	5.01E-170	chr1_000006F_(-1)	92.6	94.53	309	525.4
44	jgi|Aspni_NRRL3_1|264|	1	1.18E-66	chr1_000006F_(+2)	85.71	85.71	126	218.78
45	jgi|Aspni_NRRL3_1|329|	2	7.86E-05	chr1_000006F_(-1)	88.24	94.12	77	40.43
46	jgi|Aspni_NRRL3_1|360|	1	3.50E-60	chr1_000006F_(-3)	56.59	58.54	205	201.44
47	jgi|Aspni_NRRL3_1|383|	1	1.80E-24	chr1_000006F_(+1)	78.57	78.57	84	96.29
48	jgi|Aspni_NRRL3_1|560|	1	2.42E-88	chr1_000006F_(-2)	80.1	80.1	201	283.88
49	jgi|Aspni_NRRL3_1|739|	2	3.64E-22	chr1_000006F_(-2)	82.5	90	181	93.2
50	jgi|Aspni_NRRL3_1|840|	2	2.38E-92	chr1_000006F_(+1)	80.41	80.93	194	297.36
51	jgi|Aspni_NRRL3_1|862|	2	2.62E-108	chr1_000006F_(-1)	89.47	92.11	228	346.28
52	jgi|Aspni_NRRL3_1|910|	2	9.45E-72	chr1_000006F_(-2)	75.84	76.58	269	239.19
53	jgi|Aspni_NRRL3_1|977|	2	5.01E-28	chr1_000006F_(-1)	64.37	73.56	112	109.38
54	jgi|Aspni_NRRL3_1|1062|	2	1.50E-75	chr1_000006F_(+2)	77.71	80.25	157	245.74
55	jgi|Aspni_NRRL3_1|1145|	1	1.75E-113	chr1_000006F_(+3)	81.1	81.1	254	358.22
56	jgi|Aspni_NRRL3_1|1200|	3	8.10E-14	chr6_000005F_(-1)	58.06	61.29	468	77.03
57	jgi|Aspni_NRRL3_1|1213|	3	7.32E-18	chr3_000004F_(-1)	24.53	42.77	453	89.74
58	jgi|Aspni_NRRL3_1|1341|	2	2.24E-32	chr2_000000F_(+2)	69.23	74.04	103	121.32

59	jgi|Aspni_NRRL3_1|1344|	2	6.70E-104	chr2_000000F_(+2)	80.98	80.98	205	328.56
60	jgi|Aspni_NRRL3_1|1609|	1	4.63E-22	chr2_000000F_(+1)	54.21	59.81	107	90.51
61	jgi|Aspni_NRRL3_1|1613|	2	5.50E-64	chr2_000000F_(-1)	68.83	68.83	171	214.54
62	jgi|Aspni_NRRL3_1|1677|	1	9.35E-04	chr3_000004F_(-2)	24.11	44.68	117	43.13
63	jgi|Aspni_NRRL3_1|1680|	9	7.39E-24	chr7_000002F_(-3)	48.87	57.14	157	99.75
64	jgi|Aspni_NRRL3_1|1683|	4	9.14E-28	chr6_000005F_(+1)	91.18	91.23	57	110.54
65	jgi|Aspni_NRRL3_1|1685|	4	8.38E-33	chr5_000008F_(-1)	84.93	90.41	82	129.8
66	jgi|Aspni_NRRL3_1|1698|	1	3.08E-14	chr5_000008F_(+3)	33.88	54.55	113	73.94
67	jgi|Aspni_NRRL3_1|1773|	2	6.93E-83	chr2_000000F_(+1)	88.62	90.42	167	271.94
68	jgi|Aspni_NRRL3_1|1777|	2	2.06E-24	chr2_000000F_(+1)	64.79	67.61	71	96.67
69	jgi|Aspni_NRRL3_1|1799|	1	7.40E-03	chr2_000000F_(-1)	91.89	94.59	37	35.42
70	jgi|Aspni_NRRL3_1|1831|	2	1.04E-124	chr2_000000F_(+1)	86.09	86.52	230	390.58
71	jgi|Aspni_NRRL3_1|2275|	1	7.81E-89	chr2_000000F_(+3)	91.98	92.59	162	285.03
72	jgi|Aspni_NRRL3_1|2287|	1	2.93E-33	chr2_000000F_(+3)	58.46	60	130	122.48
73	jgi|Aspni_NRRL3_1|2323|	1	3.54E-55	chr2_000000F_(-3)	69.5	69.5	141	186.81
74	jgi|Aspni_NRRL3_1|2340|	3	5.71E-137	chr2_000000F_(+1)	89.71	90.53	254	446.82
75	jgi|Aspni_NRRL3_1|2379|	1	2.50E-62	chr2_000000F_(+1)	80.15	80.92	131	207.22
76	jgi|Aspni_NRRL3_1|2463|	2	1.40E-68	chr2_000000F_(+2)	69.35	71.51	186	229.56
77	jgi|Aspni_NRRL3_1|2594|	1	1.69E-04	chr7_000002F_(-2)	42.42	50	64	47.75
78	jgi|Aspni_NRRL3_1|2665|	1	2.47E-65	chr2_000000F_(+2)	88.89	88.89	216	219.55
79	jgi|Aspni_NRRL3_1|2730|	1	1.08E-44	chr2_000000F_(+1)	49.28	53.62	207	157.53
80	jgi|Aspni_NRRL3_1|2768|	2	1.39E-61	chr2_000000F_(-1)	83.05	83.05	118	206.07
81	jgi|Aspni_NRRL3_1|2936|	2	1.63E-141	chr3_000004F_(+1)	91.7	93.78	241	442.19
82	jgi|Aspni_NRRL3_1|3009|	1	6.03E-08	chr3_000004F_(+2)	42.5	50	78	51.99
83	jgi|Aspni_NRRL3_1|3018|	2	6.52E-87	chr3_000004F_(-3)	82.1	87.04	162	280.41
84	jgi|Aspni_NRRL3_1|3020|	2	6.37E-14	chr3_000004F_(-3)	73.47	79.59	99	71.63
85	jgi|Aspni_NRRL3_1|3048|	1	6.09E-91	chr3_000004F_(-2)	90.51	91.14	158	290.43
86	jgi|Aspni_NRRL3_1|3285|	2	1.07E-16	scaffold3_000013F_(+3)	28.57	43.21	247	85.5
87	jgi|Aspni_NRRL3_1|3286|	1	1.44E-03	chr3_000004F_(-2)	22.16	41.24	168	42.36
88	jgi|Aspni_NRRL3_1|3287|	1	8.99E-03	chr7_000002F_(-1)	41.46	63.41	39	38.12
89	jgi|Aspni_NRRL3_1|3289|	2	3.33E-06	chr2_000000F_(+1)	29.92	53.26	122	48.91
90	jgi|Aspni_NRRL3_1|3299|	2	2.06E-16	chr2_000000F_(-1)	31.87	48.35	271	85.5
91	jgi|Aspni_NRRL3_1|3305|	2	1.77E-22	scaffold1_000010F_(-2)	31.91	48.94	262	99.37
92	jgi|Aspni_NRRL3_1|3306|	1	1.65E-06	chr5_000007F_(+1)	21.51	39.62	226	53.91
93	jgi|Aspni_NRRL3_1|3312|	1	5.12E-142	chr3_000004F_(-3)	87.15	87.55	249	440.27
94	jgi|Aspni_NRRL3_1|3336|	1	1.44E-35	chr3_000004F_(+2)	79.81	79.81	104	128.64
95	jgi|Aspni_NRRL3_1|3338|	1	2.93E-24	chr3_000004F_(+2)	75	76.25	80	95.52
96	jgi|Aspni_NRRL3_1|3352|	1	1.34E-52	chr3_000004F_(-3)	81.25	82.14	112	177.95

97	jgi|Aspni_NRRL3_1|3412|	1	2.79E-35	chr3_000004F_(+1)	64.81	69.44	108	128.26
98	jgi|Aspni_NRRL3_1|3425|	1	7.37E-110	chr3_000004F_(+3)	88.42	88.95	190	345.89
99	jgi|Aspni_NRRL3_1|3462|	1	4.98E-89	chr3_000004F_(+3)	87.82	87.82	156	284.65
100	jgi|Aspni_NRRL3_1|3497|	1	7.84E-19	chr3_000004F_(-2)	65.15	66.67	66	80.88
101	jgi|Aspni_NRRL3_1|3735|	1	1.05E-68	chr3_000004F_(-2)	84.85	84.85	132	227.25
102	jgi|Aspni_NRRL3_1|3825|	1	1.49E-97	chr3_000004F_(+2)	78.78	78.78	245	312
103	jgi|Aspni_NRRL3_1|4190|	2	1.36E-92	chr3_000004F_(-3)	81.97	82.51	183	299.29
104	jgi|Aspni_NRRL3_1|4216|	2	6.66E-14	chr1_000006F_(-3)	58.14	72.09	114	68.17
105	jgi|Aspni_NRRL3_1|4416|	3	1.02E-25	chr4_000001F_(-2)	74.65	86.84	255	117.09
106	jgi|Aspni_NRRL3_1|4427|	1	3.44E-07	chr5_000007F_(+3)	43.42	51.32	75	47.37
107	jgi|Aspni_NRRL3_1|4428|	2	9.64E-08	chr1_000006F_(-3)	65	70	57	51.22
108	jgi|Aspni_NRRL3_1|4444|	1	8.37E-48	chr4_000001F_(-2)	84.95	84.95	93	163.7
109	jgi|Aspni_NRRL3_1|4446|	1	1.57E-41	chr4_000001F_(+2)	77.55	78.57	98	145.59
110	jgi|Aspni_NRRL3_1|4663|	2	8.68E-57	chr4_000001F_(+1)	93.75	93.75	143	194.9
111	jgi|Aspni_NRRL3_1|4735|	2	7.13E-11	chr4_000001F_(+1)	46	48	78	57
112	jgi|Aspni_NRRL3_1|4772|	2	5.51E-141	chr4_000001F_(-1)	70.3	70.3	330	441.43
113	jgi|Aspni_NRRL3_1|4826|	1	3.19E-84	chr4_000001F_(-3)	89.47	90.64	171	271.94
114	jgi|Aspni_NRRL3_1|4882|	1	3.55E-79	chr4_000001F_(+1)	91.75	91.75	194	258.45
115	jgi|Aspni_NRRL3_1|4903|	2	0	chr4_000001F_(+2)	90.84	93.13	655	1 199,11
116	jgi|Aspni_NRRL3_1|4911|	2	4.97E-73	chr4_000001F_(-3)	86.47	87.22	133	237.65
117	jgi|Aspni_NRRL3_1|5096|	1	4.81E-65	chr4_000001F_(-1)	85.6	85.6	125	214.16
118	jgi|Aspni_NRRL3_1|5127|	1	3.88E-82	chr4_000001F_(-2)	87.1	87.1	155	264.62
119	jgi|Aspni_NRRL3_1|5441|	2	1.98E-11	chr7_000002F_(+2)	27.39	43.98	335	69.32
120	jgi|Aspni_NRRL3_1|5446|	2	9.79E-14	chr8_000003F_(+1)	35.14	50.74	145	73.56
121	jgi|Aspni_NRRL3_1|5674|	1	8.76E-70	chr4_000001F_(+3)	80.89	80.89	157	228.79
122	jgi|Aspni_NRRL3_1|5902|	2	0	chr4_000001F_(+2)	88.68	89.1	477	816.61
123	jgi|Aspni_NRRL3_1|5964|	1	2.39E-35	chr4_000001F_(+2)	78.41	78.41	88	127.49
124	jgi|Aspni_NRRL3_1|5978|	2	7.79E-20	chr4_000001F_(-2)	75	87.5	129	83.57
125	jgi|Aspni_NRRL3_1|6180|	1	0	chr4_000001F_(-2)	88.99	88.99	345	590.11
126	jgi|Aspni_NRRL3_1|6191|	1	1.44E-55	chr4_000001F_(-3)	81.54	86.92	130	188.35
127	jgi|Aspni_NRRL3_1|6272|	1	3.31E-03	chr1_000006F_(-2)	26.86	45.71	172	39.66
128	jgi|Aspni_NRRL3_1|6274|	2	4.44E-35	chr2_000000F_(+1)	29.64	48.86	299	136.73
129	jgi|Aspni_NRRL3_1|6278|	2	1.36E-03	chr3_000004F_(-2)	39.13	63.04	47	38.89
130	jgi|Aspni_NRRL3_1|6308|	2	5.80E-59	chr5_000007F_(+3)	92.31	92.31	133	199.52
131	jgi|Aspni_NRRL3_1|6386|	2	1.95E-33	chr5_000007F_(+1)	80.9	80.9	89	122.09
132	jgi|Aspni_NRRL3_1|6407|	1	4.39E-45	chr5_000007F_(+3)	82.83	82.83	99	155.99
133	jgi|Aspni_NRRL3_1|6443|	2	3.16E-51	chr5_000007F_(+2)	53.62	60.87	214	186.04
134	jgi|Aspni_NRRL3_1|6514|	1	8.43E-54	chr5_000007F_(+3)	69.03	70.32	155	182.57

135	jgi|Aspni_NRRL3_1|6523|	1	2.39E-65	chr5_000007F_(+1)	85.48	86.29	124	215.31
136	jgi|Aspni_NRRL3_1|6550|	1	8.07E-147	chr5_000007F_(+3)	89.16	89.56	249	454.52
137	jgi|Aspni_NRRL3_1|6593|	1	2.51E-28	chr5_000007F_(-2)	71.23	71.23	73	106.3
138	jgi|Aspni_NRRL3_1|6606|	2	0	chr5_000007F_(+3)	92.59	94.44	466	697.58
139	jgi|Aspni_NRRL3_1|6827|	2	4.32E-37	chr5_000007F_(-2)	60.29	67.65	133	138.66
140	jgi|Aspni_NRRL3_1|7017|	2	7.36E-29	chr2_000000F_(-2)	63.16	68.42	107	109.38
141	jgi|Aspni_NRRL3_1|7021|	1	2.46E-80	chr2_000000F_(+1)	80.62	81.88	160	259.23
142	jgi|Aspni_NRRL3_1|7123|	2	1.18E-05	chr7_000002F_(+1)	28.57	49.58	147	52.37
143	jgi|Aspni_NRRL3_1|7129|	3	8.41E-62	scaffold2_000012F_(-2)	84.54	90.72	142	206.07
144	jgi|Aspni_NRRL3_1|7133|	1	1.33E-11	chr6_000005F_(+3)	59.68	62.9	62	63.93
145	jgi|Aspni_NRRL3_1|7134|	1	5.15E-68	chr4_000011F_(-1)	60.1	73.74	194	228.41
146	jgi|Aspni_NRRL3_1|7147|	2	3.22E-157	chr4_000011F_(-2)	85.04	90.03	362	491.12
147	jgi|Aspni_NRRL3_1|7156|	3	8.99E-33	chr4_000001F_(+2)	62.5	78.12	242	132.11
148	jgi|Aspni_NRRL3_1|7168|	2	1.94E-17	scaffold3_000013F_(-3)	33.78	53.38	150	87.04
149	jgi|Aspni_NRRL3_1|7170|	2	5.16E-66	chr5_000008F_(+2)	50.23	64.25	221	224.94
150	jgi|Aspni_NRRL3_1|7189|	3	4.40E-16	chr2_000000F_(-1)	37.36	50.61	214	79.34
151	jgi|Aspni_NRRL3_1|7193|	1	1.23E-03	chr5_000008F_(+2)	24.79	42.86	226	44.67
152	jgi|Aspni_NRRL3_1|7194|	2	1.07E-08	chr5_000008F_(+1)	48.39	62.9	62	54.3
153	jgi|Aspni_NRRL3_1|7201|	2	4.50E-09	chr5_000007F_(-1)	83.33	83.33	30	53.53
154	jgi|Aspni_NRRL3_1|7218|	1	2.72E-70	chr4_000001F_(-3)	63.13	75.76	198	235.34
155	jgi|Aspni_NRRL3_1|7223|	2	1.01E-10	chr5_000008F_(-2)	80.56	86.11	49	59.69
156	jgi|Aspni_NRRL3_1|7226|	2	4.97E-16	chr5_000008F_(-1)	38.06	47.1	149	75.87
157	jgi|Aspni_NRRL3_1|7227|	4	1.61E-14	chr5_000008F_(+3)	81.4	83.72	45	69.32
158	jgi|Aspni_NRRL3_1|7419|	3	7.06E-14	chr2_000000F_(+2)	34.88	50	245	75.1
159	jgi|Aspni_NRRL3_1|7434|	2	1.84E-38	chr6_000005F_(+1)	84.62	86.79	78	139.43
160	jgi|Aspni_NRRL3_1|7656|	1	2.55E-58	chr6_000005F_(+1)	73.38	73.38	139	194.9
161	jgi|Aspni_NRRL3_1|7820|	3	1.05E-28	chr6_000005F_(+1)	63	70.97	100	110.15
162	jgi|Aspni_NRRL3_1|7951|	1	3.58E-27	chr6_000005F_(+2)	67.57	69.37	110	106.3
163	jgi|Aspni_NRRL3_1|8016|	2	1.23E-106	chr6_000005F_(+2)	78.4	79.81	213	336.65
164	jgi|Aspni_NRRL3_1|8030|	2	6.92E-29	chr6_000005F_(-1)	53.62	57.97	128	110.92
165	jgi|Aspni_NRRL3_1|8058|	2	7.62E-25	chr6_000005F_(+3)	86.96	86.96	120	98.6
166	jgi|Aspni_NRRL3_1|8264|	1	2.58E-179	chr6_000005F_(+2)	90.85	90.85	295	549.67
167	jgi|Aspni_NRRL3_1|8620|	2	6.02E-14	scaffold1_000010F_(+3)	84.21	92.11	55	67.01
168	jgi|Aspni_NRRL3_1|8724|	1	3.94E-29	scaffold1_000010F_(-2)	68.35	69.62	79	109
169	jgi|Aspni_NRRL3_1|8924|	3	1.70E-45	chr7_000002F_(-1)	49.12	66.67	227	163.31
170	jgi|Aspni_NRRL3_1|8948|	1	1.55E-93	chr7_000002F_(-2)	89.31	91.82	159	298.52
171	jgi|Aspni_NRRL3_1|8952|	1	3.87E-77	chr7_000002F_(-2)	78.4	78.4	162	249.98
172	jgi|Aspni_NRRL3_1|9151|	1	6.08E-90	chr7_000002F_(+1)	85.57	85.57	194	288.5

173	jgi|Aspni_NRRL3_1|9306|	2	7.47E-53	chr7_000002F_(+3)	81.05	84.21	139	184.5
174	jgi|Aspni_NRRL3_1|9448|	1	0	chr7_000002F_(-1)	85.71	89.92	357	593.19
175	jgi|Aspni_NRRL3_1|9465|	2	8.22E-107	chr7_000002F_(-3)	81.11	81.57	217	337.81
176	jgi|Aspni_NRRL3_1|9468|	2	1.70E-13	chr7_000002F_(-3)	86.36	90.91	59	77.03
177	jgi|Aspni_NRRL3_1|9473|	2	4.74E-31	chr4_000011F_(-3)	66.67	80.95	113	124.02
178	jgi|Aspni_NRRL3_1|9505|	1	2.36E-77	chr7_000002F_(-1)	86.17	86.17	188	252.29
179	jgi|Aspni_NRRL3_1|9522|	2	6.63E-67	chr7_000002F_(-3)	79.41	91.91	164	229.18
180	jgi|Aspni_NRRL3_1|9524|	2	8.67E-102	chr7_000002F_(+1)	70.42	80.28	321	331.64
181	jgi|Aspni_NRRL3_1|9562|	1	1.36E-32	chr2_000000F_(-3)	33.48	51.58	220	126.33
182	jgi|Aspni_NRRL3_1|9654|	1	4.74E-19	chr2_000000F_(+3)	60.76	70.89	79	92.05
183	jgi|Aspni_NRRL3_1|9670|	2	5.84E-10	chr4_000011F_(+1)	39.78	52.69	143	62.39
184	jgi|Aspni_NRRL3_1|9705|	2	1.59E-88	chr7_000002F_(-2)	69.33	72.92	225	290.81
185	jgi|Aspni_NRRL3_1|9706|	2	7.18E-31	chr7_000002F_(+2)	76.92	89.23	65	116.7
186	jgi|Aspni_NRRL3_1|9707|	1	7.65E-03	chr1_000006F_(-1)	27	43	90	41.2
187	jgi|Aspni_NRRL3_1|9724|	2	7.68E-40	chr7_000002F_(-1)	90.91	90.91	123	142.12
188	jgi|Aspni_NRRL3_1|9812|	2	1.80E-11	chr7_000002F_(+2)	39.24	62.03	72	69.71
189	jgi|Aspni_NRRL3_1|9815|	4	4.62E-11	chr8_000003F_(-2)	48.65	70.27	275	68.55
190	jgi|Aspni_NRRL3_1|9963|	2	2.17E-38	scaffold3_000013F_(+2)	82.19	91.78	73	136.35
191	jgi|Aspni_NRRL3_1|10010|	1	2.21E-25	chr7_000002F_(-3)	92.59	94.44	54	98.6
192	jgi|Aspni_NRRL3_1|10038|	1	4.86E-43	chr7_000002F_(+1)	56.69	56.69	157	150.6
193	jgi|Aspni_NRRL3_1|10073|	4	2.00E-37	chr7_000002F_(-3)	78.79	84.85	319	146.75
194	jgi|Aspni_NRRL3_1|10310|	1	5.86E-71	chr8_000003F_(-2)	88.81	88.81	134	235.73
195	jgi|Aspni_NRRL3_1|10369|	1	6.47E-03	chr6_000005F_(-1)	27.72	36.63	189	40.82
196	jgi|Aspni_NRRL3_1|10370|	2	9.26E-05	chr2_000000F_(-3)	40.43	65.96	71	47.75
197	jgi|Aspni_NRRL3_1|10371|	3	5.68E-22	chr1_000006F_(+3)	36.84	49.62	232	97.06
198	jgi|Aspni_NRRL3_1|10372|	2	1.20E-25	chr4_000001F_(-1)	54.1	75.41	345	112.08
199	jgi|Aspni_NRRL3_1|10474|	4	2.09E-127	chr8_000003F_(+1)	80.95	86.67	271	408.68
200	jgi|Aspni_NRRL3_1|10699|	1	2.83E-117	chr8_000003F_(+1)	80.8	82.61	275	370.55
201	jgi|Aspni_NRRL3_1|10825|	1	1.82E-32	chr8_000003F_(+1)	77.92	77.92	77	119.4
202	jgi|Aspni_NRRL3_1|10888|	1	1.27E-34	chr8_000003F_(-1)	69.3	69.3	114	125.95
203	jgi|Aspni_NRRL3_1|11050|	2	1.23E-110	chr8_000003F_(+2)	83.78	86.49	260	352.44
204	jgi|Aspni_NRRL3_1|11129|	1	4.67E-12	chr8_000003F_(-1)	64.15	66.04	53	60.08
205	jgi|Aspni_NRRL3_1|11186|	1	2.86E-67	chr8_000003F_(+3)	77.55	78.23	147	221.48
206	jgi|Aspni_NRRL3_1|11373|	2	1.20E-17	chr1_000006F_(-2)	37.14	60	106	78.95
207	jgi|Aspni_NRRL3_1|11542|	2	3.08E-65	chr8_000009F_(+2)	79.1	79.85	134	214.93
208	jgi|Aspni_NRRL3_1|11547|	1	5.97E-68	chr8_000009F_(-1)	73.56	73.56	174	223.79
209	jgi|Aspni_NRRL3_1|11716|	1	2.54E-119	chr8_000009F_(+1)	66.78	69.74	304	375.94
