# Supplementary material for: Genome sequencing of the neotype strain CBS 554.65 reveals the MAT1–2 locus of Aspergillus niger
Source: BMC Genomics. 2021 Sep 21;22:679. doi: 10.1186/s12864-021-07990-8 (PMC8454179; doi:10.1186/s12864-021-07990-8)
Supplement: Supplementary file 7 — Additional file 7: Table S8. Lenght of the contigs of CBS 554.65 and coordinates of the NRRL3 and CBS 513.88 contig alignments to CBS 554.65. [file 12864_2021_7990_MOESM7_ESM.pdf]

Table S8. Lenght of the contigs of CBS 554.65 and coordinates of the NRRL3 and CBS 513.88 contig alignments to CBS 554.65.

Contig CBS 554.65	Contig lenght	Coordinates NRRL3 contig alignments to CBS 554.65			Coordinates CBS 513.88 contig alignments to CBS 554.65		
chr1_000006F	3.471.627	chr_102: 1.742.060 – 9.487	chr_101: 1.627.196 – 22.823		An14: 1.726.705 – 1	An09: 1.595.066 – 8.938	
chr2_000000F	6.394.477	chr_202: 3.472.469 – 1	chr_201: 1.114.990 – 57.501	chr_502: 999.676 – 2.458.514	An01: 3.309.842 – 6.262	An13: 1 – 908.643	An16: 1.176.026 – 1
chr3_000004F	3.949.361	chr_301: 1.795 – 1.980.559	chr_302: 1 – 1.970.163		An12: 2.565.867 – 888.334	An05: 257.064 - 1	An15: 1 – 1.854.560
chr4_000001F	6.155.019	chr_401: 580.080 – 2.466.009	chr_402: 1 – 3.754.826		An07: 492.024 – 2.341.370	An02: 3.607.648 – 790.809; 781.426 – 8.830	
chr4_000011F	789.041	chr_601: 608.795 – 529.564	chr_401: 518.476 – 74		An04: 1.903.500 – 1.974.078	An07: 479.863 – 145.235	
chr5_000007F	2.292.489	chr_501: 22.332 – 92.8111	chr_502: 4.662 – 999.412		An10: 1 – 260.207	An17: 1 – 594.595	An16: 2.132.688 – 1.176.017
chr5_000008F	1.923.516	chr_202: 3.771.556 – 3.472.459			An01: 3.625.451 – 3.321.946		
chr6_000005F	3.942.762	chr_601: 19.041 – 3.570.592			An04: 2.525.243 - 1	An03: 1.609.811 – 632.423	
chr7_000002F	4.237.642	chr_701: 21.402 – 1.233.765	chr_702: 1 – 2.792.268		An05: 568.808 – 343.165; 262.078 – 343.169	An12: 888.336 – 1	An11: 2.721.467 – 1
chr8_000003F	4.067.305	chr_802: 2.494.337 – 82.862	chr_801: 1.604.814 – 1.465		An08: 2.392.212 - 1	An18: 1.542.301 – 1	
chr8_000009F	930.410	chr_802: 2.494.842 – 3.394.669			An08: 2.392.761 – 2.567.719; 2.647.254 – 2.567.718	An06: 490.392 – 20.556; 490.393 – 681.473	
scaffold1_000010F	760.296	chr_601: 4.257.816 – 3.621.589			An03: 1 – 638.135		
scaffold2_000012F	658.424						
scaffold3_000013F	504.300						
scaffold4_000014R	209.345						
scaffold5_000015F	75.111	chr_601: 3.623.696 – 3.628.443			An03: 636028 - 639267		
scaffold6_000016F	64.108	chr_601: 3.621.444– 3.629.595			An03: 643987 - 647696		
