# Supplementary material for: Genome sequencing of the neotype strain CBS 554.65 reveals the MAT1–2 locus of Aspergillus niger
Source: BMC Genomics. 2021 Sep 21;22:679. doi: 10.1186/s12864-021-07990-8 (PMC8454179; doi:10.1186/s12864-021-07990-8)
Supplement: Supplementary file 8 — Additional file 8: Table S9. Gene identifiers of the analyzed Aspergillus strains and their position in the MAT locus. [file 12864_2021_7990_MOESM8_ESM.pdf]

Table S9. Gene identifiers of the analyzed Aspergillus strains and their position in the MAT locus.

Section	Species	Strain	Conserved gene left flank SAICAR synthetase - ad50	DNA lyase - apnB	Citochrome c oxidase - coxM	Anaphase-promoting complex protein - apcE	Conserved gene right flank Cytoskeleton assembly control protein - slaB	Mating-type gene - MAT	Mating-type	MAT position	Sexual cycle described for the species
Nigri	*A. welwitschiae*	CBS 139.54	147365	Not annotated	147372	172184	Not annotated	172181	MAT1-1	flipped	No
	A. kawachii	IFO 4308	AKAW_03838	AKAW_03833	AKAW_03834	AKAW_03835	AKAW_03826	AKAW_03832	MAT1-2	conserved	No
	A. luchuensis	106.47	ASPFODRAFT_203813	ASPFODRAFT_123916	ASPFODRAFT_41814	ASPFODRAFT_41813	ASPFODRAFT_203824	ASPFODRAFT_180958	MAT1-1	conserved	No
	A. tubingensis	G131	Not annotated	Not annotated	Not annotated	Not annotated	Not annotated	Not annotated	MAT1-2	conserved	Yes [1]
		CBS 134.48	ASPTUDRAFT_932672	ASPTUDRAFT_44744	ASPTUDRAFT_174406	ASPTUDRAFT_717678	ASPTUDRAFT_67364	ASPTUDRAFT_124452	MAT1-1	conserved	
	A. niger	CBS 554.65	g9049	g9042	g9043	g9044	g9036	g9041	MAT1-2	conserved	No
		ATCC 1015	ASPNIDRAFT2_1225150	ASPNIDRAFT2_1187921	ASPNIDRAFT2_1147272	ASPNIDRAFT2_1187923	ASPNIDRAFT_1128148	ASPNIDRAFT2_1178859	MAT1-1	flipped	
	A. brasiliensis	CBS 101740	ASPBRDRAFT_265564	ASPBRDRAFT_37526	ASPBRDRAFT_112056	ASPBRDRAFT_114297	ASPBRDRAFT_61705	ASPBRDRAFT_167991	MAT1-2	flipped	No
	A. carbonarius	ITEM 5010	ASPCADRAFT_204319	ASPCADRAFT_204314	ASPCADRAFT_204313	ASPCADRAFT_139768	n.a[2]	ASPCADRAFT_1991	MAT1-2	conserved	No
	A. aculeatus	ATCC 16872	ASPACDRAFT_77574	ASPACDRAFT_117017	ASPACDRAFT_42175	ASPACDRAFT_27181	ASPACDRAFT_117010	ASPACDRAFT_1867751	MAT1-2	conserved	No
Versicolores	*A. versicolor*	CBS 583.65	ASPVEDRAFT_40204	ASPVEDRAFT_82220	ASPVEDRAFT_51798	ASPVEDRAFT_40208	ASPVEDRAFT_40212	ASPVEDRAFT_82222	MAT1-2	conserved	No
	A. sydowii	CBS 593.65	ASPSYDRAFT_147430	ASPSYDRAFT_87882	ASPSYDRAFT_146414	ASPSYDRAFT_56669	ASPSYDRAFT_43291	ASPSYDRAFT_87884	MAT1-2	conserved	No
Ochraceorosei	*A. ochraceoroseus*	IBT 24754	P175DRAFT_0556899	P175DRAFT_0477718	P175DRAFT_0434832	P175DRAFT_0457183	P175DRAFT_0477742	P175DRAFT_0477739	MAT1-1	conserved	No
Flavi	*A. flavus*	NRRL 3357	AFLA_100180	AFLA_103190	AFLA_103180	AFLA_103170	AFLA_103220	AFLA_103210	MAT1-1	conserved	Yes [2]
	A. oryzae	BCC7051	OAory_01098770	OAory_01101290	OAory_01101280	OAory_01101270	OAory_01101320	OAory_01101300	MAT1-2	conserved	No
		RIB40	AO090020000395	AO090020000091	n.a.[2]	AO090020000092	AO090020000087	AO090020000089	MAT1-1	conserved	
Circumdati	*A. steynii*	IBT 23096	P170DRAFT_402429	P170DRAFT_461838	P170DRAFT_461837	P170DRAFT_453296	P170DRAFT_376660	P170DRAFT_349471	MAT1-2	conserved	No
Candidi	*A. campestris*	IBT 28561	P168DRAFT_228536	P168DRAFT_285959	P168DRAFT_300586	P168DRAFT_308029	P168DRAFT_285953	P168DRAFT_313902	MAT1-1	conserved	No
								P168DRAFT_285957	MAT1-2	conserved	
Terrei	*A. terreus*	NIH2624	ATEG_08807	ATEG_08811	ATEG_08810	ATEG_08809	ATEG_08813	ATEG_08812	MAT1-1	conserved	Yes [3]
Fumigati	*A. novofumigatus*	IBT 16806	P174DRAFT_410276	P174DRAFT_443610	P174DRAFT_244239	P174DRAFT_392973	P174DRAFT_443607	P174DRAFT_462167	MAT1-2	conserved	No
	A. fischeri	NRRL 181	NFIA_071050	NFIA_071080	NFIA_071070	NFIA_071060	NFIA_071110	NFIA_071100	MAT1-1	conserved	Yes [4]
								NFIA_024390	MAT1-2	conserved	
	A. fumigatus	Af293	Afu3g06210	Afu3g06180	Afu3g06190	Afu3g06200	Afu3g06140	Afu3g06170	MAT1-2	conserved	Yes [5]
		A1163	AFUB_042850	AFUB_042880	AFUB_042870	AFUB_042860	AFUB_042910	AFUB_042900	MAT1-1	conserved	
								AFUB_042890	MAT1-2	conserved	
Clavati	*A. clavatus*	NRRL1	ACLA_034160	ACLA_034130	ACLA_034140	ACLA_034150	ACLA_034100	ACLA_034110	MAT1-1	conserved	Yes [6]
								ACLA_034120	MAT1-2	conserved	
Aspergillus	*A. glaucus*	CBS 516.65	ASPGLDRAFT_126756	n.a.[2]	ASPGLDRAFT_149347	ASPGLDRAFT_126686	ASPGLDRAFT_74309	ASPGLDRAFT_89185	MAT1-1	n.a.[1]	Yes [7,8]
Cremei	*A. wentii*	DTO 134E9	ASPWEDRAFT_69948	ASPWEDRAFT_42165	ASPWEDRAFT_565475	ASPWEDRAFT_60656	ASPWEDRAFT_42170	ASPWEDRAFT_184745	MAT1-2	conserved	No

[1] Conserved genes not in the MAT locus

[2] Gene not found

[1] Horn BW, Olarte RA, Peterson SW, Carbone I. Sexual reproduction in *Aspergillus tubingensis* from section *Nigri*. Mycologia 2013;105:1153–63. https://doi.org/10.3852/13-101.

[2] Horn BW, Moore GG, Carbone I. Sexual reproduction in *Aspergillus flavus*. Mycologia 2009;101:423–9. https://doi.org/10.3852/09-011.

[3] Arabatzis M. Sexual reproduction in the opportunistic human pathogen *Aspergillus terreus* 2013;105:71–9. https://doi.org/10.3852/11-426.

[4] Raper KB, Fennell DI. The genus *Aspergillus*. 1965.

[5] O'Gorman CM, Fuller HT, Dyer PS. Discovery of a sexual cycle in the opportunistic fungal pathogen *Aspergillus fumigatus*. Nature 2009;457:471–4. https://doi.org/10.1038/nature07528.

[6] Ojeda-López M, Chen W, Eagle CE, Gutiérrez G, Jia WL, Swilaiman SS, et al. Evolution of asexual and sexual reproduction in the aspergilli. Stud Mycol 2018;91:37–59. https://doi.org/10.1016/j.simyco.2018.10.002.

[7] Link HF. Observationes in ordines plantarum naturales. Dissertatio I. Mag Ges Naturf Freunde Berlin 3 1809;3–42.

[8] Chen AJ, Hubka V, Frisvad JC, Visagie CM, Houbraken J, Meijer M, et al. Polyphasic taxonomy of *Aspergillus* section *Aspergillus* (formerly *Eurotium*), and its occurrence in indoor environments and food. Stud Mycol 2017;88:37–135. https://doi.org/10.1016/j.simyco.2017.07.001.
